# A common copy-number breakpoint of *ERBB2 *amplification in breast cancer colocalizes with a complex block of segmental duplications

**DOI:** 10.1186/bcr3362

**Published:** 2012-11-26

**Authors:** Michael Marotta, Xiongfong Chen, Ayako Inoshita, Robert Stephens, G Thomas Budd, Joseph P Crowe, Joanne Lyons, Anna Kondratova, Raymond Tubbs, Hisashi Tanaka

**Affiliations:** 1 Department of Molecular Genetics, Cleveland Clinic Lerner Research Institute, Cleveland, OH 44195, USA; 2 Taussig Cancer Institute, Cleveland Clinic, Cleveland, OH 44195, USA; 3 Department of General Surgery, Cleveland Clinic, Cleveland, OH 44195, USA; 4 Advanced Biomedical Computing Center, SAIC-Frederick, Inc., National Cancer Institute at Frederick, Frederick, MD 21702, USA; 5 Department of Molecular Pathology, Cleveland Clinic, Cleveland, OH 44195, USA; 6 Department of Molecular Medicine, Cleveland Clinic Lerner College of Medicine of Case Western Reserve University, Cleveland, OH 44195, USA

## Abstract

**Introduction:**

Segmental duplications (low-copy repeats) are the recently duplicated genomic segments in the human genome that display nearly identical (> 90%) sequences and account for about 5% of euchromatic regions. In germline, duplicated segments mediate nonallelic homologous recombination and thus cause both non-disease-causing copy-number variants and genomic disorders. To what extent duplicated segments play a role in somatic DNA rearrangements in cancer remains elusive. Duplicated segments often cluster and form genomic blocks enriched with both direct and inverted repeats (complex genomic regions). Such complex regions could be fragile and play a mechanistic role in the amplification of the *ERBB2 *gene in breast tumors, because repeated sequences are known to initiate gene amplification in model systems.

**Methods:**

We conducted polymerase chain reaction (PCR)-based assays for primary breast tumors and analyzed publically available array-comparative genomic hybridization data to map a common copy-number breakpoint in *ERBB2*-amplified primary breast tumors. We further used molecular, bioinformatics, and population-genetics approaches to define duplication contents, structural variants, and haplotypes within the common breakpoint.

**Results:**

We found a large (> 300-kb) block of duplicated segments that was colocalized with a common-copy number breakpoint for *ERBB2 *amplification. The breakpoint that potentially initiated *ERBB2 *amplification localized in a region 1.5 megabases (Mb) on the telomeric side of *ERBB2*. The region is very complex, with extensive duplications of *KRTAP *genes, structural variants, and, as a result, a paucity of single-nucleotide polymorphism (SNP) markers. Duplicated segments are varied in size and degree of sequence homology, indicating that duplications have occurred recurrently during genome evolution.

**Conclusions:**

Amplification of the *ERBB2 *gene in breast tumors is potentially initiated by a complex region that has unusual genomic features and thus requires rigorous, labor-intensive investigation. The haplotypes we provide could be useful to identify the potential association between the complex region and *ERBB2 *amplification.

## Introduction

Gene amplification is a cellular process characterized by a selective increase of a particular genomic region without a proportional increase of the entire genome [1-4]. The selective increase accompanies the overexpression of a particular gene within the genomic region that confers a growth advantage to the cell. The growth advantage derived from gene amplification has long been recognized as an important problem for cancer patients. Increased copy numbers of proto-oncogenes, such as *MYC*, *MYCN*, and *ERBB2*, leads to the overexpression of oncogene products that drives abnormal cell proliferation [5-9]. Abnormal cell proliferation results in cancer progression and poor patient survival [10,11]. In addition, gene amplification is an underlying mechanism for acquired therapy resistance, as cancer cells counteract therapeutic agents by overactivating either therapy-target genes (for example, *BCR-ABL *amplification) or alternative survival pathways (for example, *MET *amplification) [12-17]. Despite these adverse effects on survival of cancer patients, little is known about amplification mechanisms, and in particular, about the initiating processes of gene amplification.

During the processes of gene amplification, extra copies of large genomic segments accumulate in a cell. The accumulation could be initiated either (a) by aberrant recombination that results in the unequal distribution of chromosomal materials between daughter cells [18-22] or (b) by the loss of DNA-replication control that leads to the extra round of segmental DNA replication within a single cell cycle [23-25]. In normal cells, these processes are tightly regulated and are less likely to initiate gene amplification [26,27]. In contrast, cancer cells often lack these controls and could initiate the processes. Furthermore, cellular surveillance systems (checkpoints) that ensure genome integrity at several stages of the cell cycle are impaired in cancer cells [28,29] and could fail to eliminate cells with extra copies. Once the accumulation is initiated, it could lead to further accumulation by the growth advantage conferred by the amplified gene(s). Therefore, defining initiating processes is the key for the better understanding of the amplification mechanism. However, defining initiation processes in tumors *in vivo *is not an easy task, as current methods for evaluating gene amplification may not be feasible for capturing the amplification mechanism. Gene amplification has been measured as the increase of copy-numbers of particular genomic regions by array-comparative genomic hybridization (array-CGH) [30,31]. Although array-CGH covers the entire genome and identifies amplified regions that are important for tumor phenotypes with high confidence, such highly amplified regions may not be the initiating regions but rather the end products of adaptive evolution of cancer genomes. Next-generation sequencing could provide both copy-number profiles and somatic breakpoint sequences in cancer genomes [32,33]. Because of the copy-number increases, breakpoint sequences tend to be biased toward amplified regions and may represent late events during amplicon formation.

The difficulty in identifying initiation processes in tumors *in vivo *is typified by the *ERBB2 *amplification in breast cancer [34,35]. *ERBB2 *(v-erb-b2 erythroblastic leukemia viral oncogene homolog 2) encodes an epidermal growth-factor receptor HER2 (human epidermal growth factor receptor 2) and is amplified in 10% to 20% of invasive breast tumors [5,11]. As increased HER2 protein stimulates growth-factor signaling pathway and drives cell proliferation, *ERBB2*-amplified (HER2-positive) tumors are associated with advanced stages, recurrence, and poor patient survival [36,37]. Although the clinically significant phenotype has been known for more than two decades, the amplification mechanism remains elusive. Such information could be important for the better understanding of the etiology of *ERBB2*-amplified tumors and may have implications in future clinical practice. *ERBB2-*amplified tumors have been treated with the monoclonal antibody trastuzumab [38]. Trastuzumab binds to HER2 and downregulates growth signaling and thus has significantly improved treatment outcomes for patients with HER2-positive tumors [39-41]. An accurate diagnosis of *ERBB2 *amplification is critical, because trastuzumab is solely designed (and effective) only for tumors with *ERBB2 *amplification. Not only the mechanism of action, but also fatal cardiac side effects [42,43] and high costs (more than $100,000/year per patient) [44-46] indicate the necessity of accurate diagnosis. Currently, fluorescence *in situ *hybridization (FISH) and immunohistochemistry (IHC) are two major diagnostic tests for identifying responders and nonresponders to trastuzumab [47]. However, these current diagnostic tests have some issues, including variable results between institutions and ambiguous diagnoses, such as "equivocal" in IHC [48,49]. Preanalytic factors, such as the processing of specimens, the fixation method, and the choice of antibodies also introduce variability [50].

Amplification mechanisms could provide new information that may be useful to clarify issues associated with current tests. *ERBB2 *amplification occurs as the amplification of a genomic region surrounding *ERBB2*. A particular haplotype within the region may be more susceptible to *ERBB2 *amplification than other haplotypes. In this scenario, defining haplotypes by using patients' normal DNA could help to clarify ambiguous cases. From the tumor-biology point of view, it is not known why a subset of tumors develops *ERBB2 *amplification. For example, according to the cell-of-origin model [51], only a subset of breast tumors derived from luminal progenitor cells is HER2 positive. A better understanding of the amplification mechanism could tell us whether the lineage determination is random or has any genetic basis.

To understand the initiating mechanisms of *ERBB2 *amplification, we took integrated genomic, molecular, and bioinformatic approaches. Array-CGH data indicated that *ERBB2*-amplified tumors showed a unique pattern of copy-number transitions [52] that could result from a specific amplification mechanism (breakage-fusion- bridge (BFB) cycles). By using the BFB cycles as a guide, we identified a genomic region that could initiate *ERBB2 *amplification. The region displays a large (300-kb), complex block of duplicated segments (sequence similarity ≥ 90%) and several deletion polymorphisms. Such repeated sequences could be important in the initiation of *ERBB2 *amplification, as it has been observed in model systems that the frequency of gene amplification is shown to be determined by the presence of repeated sequences at the recombination sites [53-55]. Deletion polymorphisms of such repeated sequences may affect the initiation, and thus the frequency of *ERBB2 *amplification. Our results indicate an important role of a complex genomic region in the etiology of primary breast tumors.

## Materials and methods

### Ethics statement

This study was approved under the Cleveland Clinic internal Institutional Review Board (IRB07-136: EXEMPT: Chromosome Breakage and DNA Palindrome Formation). Specimens were obtained under the auspices of IRB 7881 (Evaluation of Genetic and Molecular Markers in Patients with Breast Cancer). All patients consented to allow their cancer specimens to be used by researchers in an anonymized fashion. The consent form indicates that publication will take place without identifiers to protect the identity of any specific individual.

### Samples and DNA extraction

Breast cancer tissues were obtained from the tissue archives in the Pathology Department, specifically from consenting patients (IRB 7881). HER2 status of these tumors was determined with FISH. We first examined hematoxylin/eosin (HE)-stained sections and confirmed that at least 80% of cellularities were derived from tumors. Five 10-mm sections were subject to DNA extraction.

Noncancerous normal DNA (HapMap DNA samples) was obtained from the Coriell Institute. The sample ID is listed in Additional file 1, Table S4.

To extract DNA, tissue sections were incubated in the lysis buffer (100 m*M *NaCl/10 m*M *Tris⋅HCl, pH 8.0/25 m*M *EDTA/0.5% SDS/proteinase K) for 24 hours at 37°C, followed by phenol/chloroform extraction and ethanol precipitation, as described previously [54].

### Array-CGH data analysis

Array-CGH datasets for 200 Her2-positive breast tumors and control normal samples (GSE21259) [52] were obtained from Gene Expression Omnibus (GEO) repository in the National Center for Biotechnology Information (NCBI) website. Partek Genomics Suite (Partek) was used to analyze the data. Raw data were normalized by using the Robust Multi-Array Average (RMA) method. RMA consists of three steps: a background adjustment, quantile normalization, and final summary. Normalized data were used to calculate the copy number of chromosome 17 in breast tumors.

### Real-time polymerase chain reaction

We used real-time PCR for measuring copy numbers in genomic DNA. Primers were designed for repeat-masked sequences of the human genome (hg18) by using MacVector (see Additional file 1, Table S5). We designed primers that amplify 100- to 200-bp genomic regions. Light Cycler 480 (Roche, Indianapolis, IN, USA) was used for real-time PCR.

For primer sets of *ERBB2*, 1, 2, 4, 5, 7, and *H19*, PCR reactions were carried out in a three-step 40-cycle reaction of 95°C for 30 seconds, 60°C for 3 seconds, and 72°C for 30 seconds by using iQ SYBR Green Supermix (Bio-Rad, Hercules, CA, USA). For primer sets of 3, 6, and 8, reactions were carried out in a two-step 40-cycle reaction of 95°C for 15 seconds and 60°C for 60 seconds. We used 5 ng/μl of genomic DNA for each reaction. Each sample was run in triplicate and was normalized to the internal control of *H19 *on chromosome 11. The primers used for this analysis are described in Additional file 1, Table S5.

### *In silico *analysis of duplication contents within the complex genomic region

A 400-kb region of chromosome 17 (36,350,000 to 36,750,000 in the human genome (hg18)) was divided into 500-bp segments (see Additional file 1, Table S1). Each segment was scanned for similar regions throughout the human genome with BLAT at the UCSC Genome Browser. To exclude the possibility of missing some of the duplicated segments that are located at the boundary of the 500-bp window, we rescanned the region by using a 2,000-bp window. We used similar criteria of sequence homology > 90%, size > 100 bp, to define (a) intraregional duplications (duplications within the 400-kb region), (b) intrachromosomal duplications (duplications between the 400-kb region and somewhere in chromosome 17 other than the 4,000-kb region), and (c) interchromosomal duplications (duplications between the 400-kb region and somewhere in the human genome other than chromosome 17).

For segments that showed similarity within the 400-kb region, a line was drawn for connecting the two fragments. Each line corresponded to a 500-bp region that includes the segment (sequence homology > 90%, size > 100 bp) mapping to another 500-bp region within the region.

Segments with > 90% homology and > 100 bp were further separated into subclassifications based on the sequence similarities and sizes. We binned the size of segments into seven groups (100 to 500 bp, 501 to 1,000 bp, 1,001 to 1,500 bp, 1,501 to 2,000 bp, 2,001 to 2,500 bp, 2,501 to 3,000 bp, and > 3,000 bp). After separating fragments into different-size bins, we defined the degree of sequence homology for each segment.

### Deletion polymorphism and PCR genotyping assay

In total, 83 structural variants were found in the Database of Genomic Variants (DGV) [56] over a 350-kb region (36.35 to 36.7 Mb). These variants were characterized by a number of different studies by using a variety of different assays (microarrays and deep sequencing) and different numbers of samples (from one individual to HapMap population). Two studies determined genotypes of structural variants for three major HapMap populations [57,58]. Only one deletion polymorphism had a minor allele frequency > 5%.

To obtain genotypes for the deletion polymorphism, two independent primer sets were designed for amplifying either the deletion allele or the nondeletion allele (see Additional file 1, Table S5). PCR was carried out in a final reaction volume of 50 μl with 1.0 U *Taq *polymerase (GoTaq, Promega, Madison, WI, USA), 1.5 m*M *MgCl_2_, 200 μ*M *dNTPs, 10 pmol of each primer, and 100 ng of genomic DNA. The thermal-cycling conditions used for amplification consisted of an initial denaturation step at 95°C for 2 minutes, followed by 30 cycles of denaturation at 95°C for 30 seconds, annealing at 60°C for 30 seconds, and extension at 72°C for 30 seconds.

### Repeated masked sequences

To determine whether "high-copy" repetitive elements are enriched within the complex genomic region, we scanned a 3-Mb region of chromosome 17: 35,000,000 to 38,000,000 (hg18) by Repeat Masker. Repeat Masker identifies interspersed repeats and low-complexity DNA and annotates these repeats into classes: SINE, LINE, LTR DNA elements, low complexity, small RNA, simple repeats, and unclassified. We binned the 3-Mb sequence into sixty 50-kb regions and made a summary of the total bp composition of each element (see Additional file 1, Figure S1).

### Linkage disequilibrium analysis

We used the HapMap SNP genotyping data (from Release 28 of International HapMap project) for three population sets: CEU, YRI, and CHB plus JPT. We took all SNP genotypes from chromosome 17: 36,350,000 to 36,800,000. To determine linkage disequilibrium between SNPs and the deletion polymorphism, we incorporated the genotype of deletion polymorphism (CNVR7096.1) from the study by Conrad *et al. *[58]. For convenience, we converted the genotypes of 0 (homozygous deletion), 1 (heterozygous), and 2 (homozygous nondeletion) to a format that could be incorporated into our existing snp data by assigning 0 to AA, 1 to AG, and 2 to GG. We incorporated the converted Conrad genotype data into the HapMap release 28 data and excluded (a) individuals from Release 28 that had not been genotyped for the CNVR7096.1 and (b) individuals for whom more than 50% of SNP genotypes were not determined. That left us with 178 YRI, 174 CEU, and 86 CHB+JPT individuals. *D'*, *LOD*, and *r^2 ^*values were calculated by using Haploview 4.2 [59].

Triangular plots were generated by using Haploview 4.2. Currently Haploview 4.2 does not support the most recent release (number 28). The previous rerelease (number 27) does have a paucity of SNPs for the 110-kb region within the complex genomic region, and we cannot generate a triangular blot for the entire region. Therefore, to include the SNPs from the release 28 into Haploview, we used SNP tools of Microsoft Excel [60] to convert the genotypes into a .ped file and .map file that are recognized by Haploview.

## Results

A series of recombination events from a single break could establish the gradient of copy-number increase toward *ERBB2*

The *ERBB2 *gene is located at chromosome 17q11.2-12. Previous studies have shown that the amplified regions (*ERBB2 *amplicon) reside within chromosomes as homogeneously staining regions (HSRs) [61-65], but not in extrachromosomal, double-minute chromosomes (DMs). Deletions of the telomeric side of *ERBB2 *are common [66,67], indicating the involvement of DNA breaks in the *ERBB2 *amplification. A large genomic region surrounding the *ERBB2 *gene is amplified, and within the amplified region, *ERBB2 *is located in the most highly amplified segment [52,68,69]. Copy number decreases gradually as it goes farther from *ERBB2*, and ends as copy-number loss (a gradient of copy-number increase). Therefore, elucidating underlying mechanisms (a) for the intrachromosomal amplification and (b) for the gradient of copy-number increase could lead to the better understanding of the mechanism of *ERBB2 *amplification.

**Figure 1:**
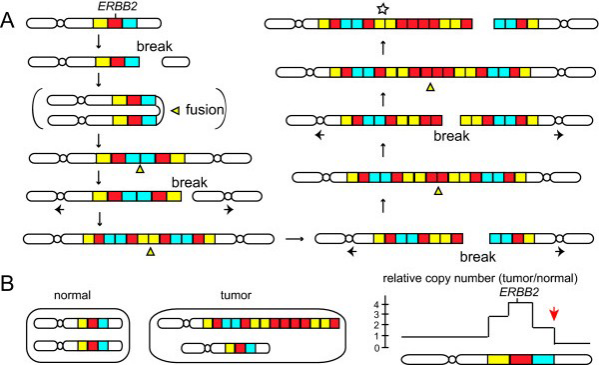
**The breakage-fusion-bridge (BFB) cycles in chromosome 17 create the gradient of copy-number increases for *ERBB2 *amplification (model)**. **(A) **A break at the telomeric side of the *ERBB2 *gene can initiate the BFB cycles and can result in *ERBB2 *amplification. Genomic segments harboring the *ERBB2 *gene are shown in red; the flanking centromeric segment is shown in yellow; and a telomeric fragment is shown in blue. In this figure, the initiating break between the blue and the white segments leads to a series of chromatid fusions and inverted duplications (centers shown in yellow triangles) that results in a chromosome with the amplified *ERBB2 *gene (star). **(B) **The BFB cycles can result in the gradient of copy-number increases on an array-CGH platform. Illustrated are a normal cell with two normal chromosomes and a tumor cell with a chromosome generated by the BFB cycles (star in A) and a normal chromosome. An array-CGH experiment for measuring relative copy number (tumor/normal) shows the gradient of copy-number increase toward the *ERBB2 *gene in the tumor cell (right). Red arrow, the copy-number transition that marks the initiating region of *ERBB2 *amplification.

One mechanism underlying intrachromosomal amplification is a well-established amplification mechanism called the breakage-fusion-bridge (BFB) cycle. The BFB cycle consists of a series of recombination events and is initiated by a chromosome break (Figure 1A) [18,19,22,70]. The replication of a broken chromosome would lead to a chromosome structure called sister chromatid fusion, in which sister chromatids are fused at a broken end. The resulting chromosome with two centromeres will have another chromosome break when two centromeres segregate into different daughter nuclei. Such a break could be resolved into sister-chromatid fusion and would initiate another round of a break and fusion. Therefore, the BFB cycles could result in the accumulation of genomic segments within the chromosome.

The accumulation of genomic segments by the BFB cycles could result in the gradient of copy-number increase (Figure 1B). An initial break could occur at the telomeric side (a blue segment) and lead to the formation of a dicentric chromosome. In the following cycle, a chromosome break at the centromeric side (a yellow segment) would be resolved into another dicentric chromosome. Further duplications and breaks would create a chromosome that accumulates segments within the chromosome. A chromosome having a segment harboring *ERBB2 *(a red segment) at very high copy number (Figure 1A, marked by a star) could be favored because of the growth advantage from *ERBB2 *overexpression. In such a chromosome, genomic segments flanking the *ERBB2*-harboring segment would also accumulate; however, because the flanking segments do not confer a growth advantage, their copy number would not be as high as that of the *ERBB2-*harboring segment. As a result, copy-number analysis for such a chromosome would show the different degree of copy-number increases between segments, and the highest increase would be seen for the segment harboring *ERBB2 *(Figure 1B). Importantly, such a scenario could predict a copy number transition for the initiating region of the BFB cycles. The initiating region (next to the blue segment) is marked by the transition from the copy-number loss to the low-level copy-number gain (red arrow) and is situated on the telomeric side of the *ERBB2 *gene.

### A common copy-number breakpoint of *ERBB2 *amplicon

**Figure 2:**
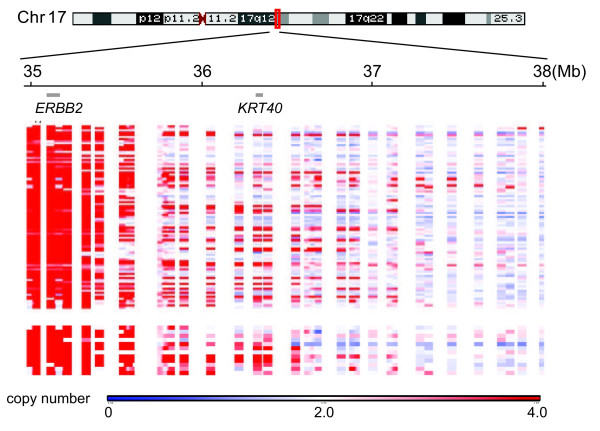
**A common copy-number breakpoint near the *KRT40 *gene**. Heat maps were created from the tiling-path BAC array data by Staaf *et al. *[52] and are shown for a 3-Mb region (35 to 38 Mb in hg18) of chromosome 17. The locations of *ERBB2 *and *KRT40 *are shown. Top, heat maps of 200 *ERBB2*-amplified breast tumors, and bottom, heat maps of a subset (11) of tumors with copy-number breakpoints near *KRT40*. Red, copy-number increase; blue, copy-number loss.

Where is the copy-number transition from a loss to a low-level gain for the *ERBB2 *amplicon? Although capturing low-level amplification is not as easy as detecting highly amplified regions with array-CGH, several studies have described such regions as the boundaries of the *ERBB2 *amplicon. For example, Sircoulomb *et al. *[71] analyzed 54 *ERBB2*-amplified breast tumors by using high-density array-CGH microarray and showed that a common telomeric boundary was predicted to be near the *KRT40 *(keratin 40) gene. The region was also described in another study as the boundary among *ERBB2*/*TOP2A *co-amplified tumors [72]. To determine whether the *KRT40 *region exhibits a common copy-number breakpoint, we analyzed a publically available array-CGH dataset that was obtained from 200 *ERBB2*-amplified tumors by using tiling-path BAC arrays (Figure 2) [52]. In the dataset, most of the tumors undergo copy-number transition from a high-level copy-number gain (*ERBB2 *region, showing in red) to a loss (regions in blue) within a 3-Mb region (chr17:35-Mb to 38-Mb in hg18) (Figure 2, top). Some tumors clearly show the copy-number transition from a gain to a loss near the *KRT40 *gene (Figure 2, bottom).

**Figure 3:**
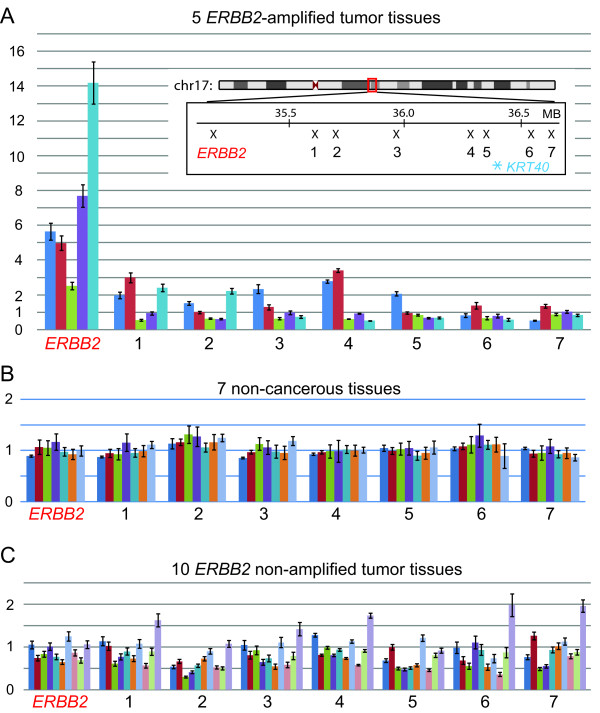
**Real-time polymerase chain reaction (PCR)-based copy-number measurements for the telomeric half of the *ERBB2 *amplicon**. Copy-number transitions for five *ERBB2*-amplified tumors **(A)**, seven normal DNA samples from HapMap individuals **(B)**, and 10 *ERBB2-*nonamplified tumors **(C) **are shown. Each color represents a copy-number transition of an individual tumor (or HapMap DNA in B). Note that two *ERBB2*-amplified tumors (blue and red in A) have a copy-number breakpoint near the *KRT40 *gene.

We confirmed the copy-number transition in the subset of *ERBB2*-amplified tumors independently by using real-time (quantitative) PCR. We designed an eight-PCR primer set for copy-number measurements within the 1.5-Mb region of the telomeric side of the *ERBB2 *gene (Figure 3A). In particular, we measured copy numbers by using four primer sets for the 370-kb region surrounding *KRT40*. To develop a sensitive and specific assay, PCR conditions and primers were optimized to provide copy numbers that were nearly equal to 1 in seven normal HapMap DNA samples (Figure 3B). Fifteen breast-tumor tissues in which *ERBB2 *amplification was determined either as *ERBB2*-positive or -negative with FISH were subject to the copy-number measurements. Consistent with the diagnoses with FISH, *ERBB2 *copy number remained low in 10 *ERBB2*-negative (by FISH) breast tumors (Figure 3C). In contrast, all five *ERBB2*-positive tumors showed copy-number increases for the *ERBB2 *gene (2.3- to 14-fold). Copy number decreased dramatically within the 500-kb region between *ERBB2 *and the primer set 1; however, two tumors (red and blue) had a low-level copy-number gain up to the region surrounding *KRT40*. In both cases, copy number decreased to one or less within the 370-kb region.

These results imply that a common copy-number breakpoint for *ERBB2 *amplification resides in the region near the *KRT40 *gene. Such a breakpoint between the copy-number gain and loss could possibly be an initiating region for *ERBB2 *amplification.

### A large block of duplicated segments at the common copy-number breakpoint

**Figure 4:**
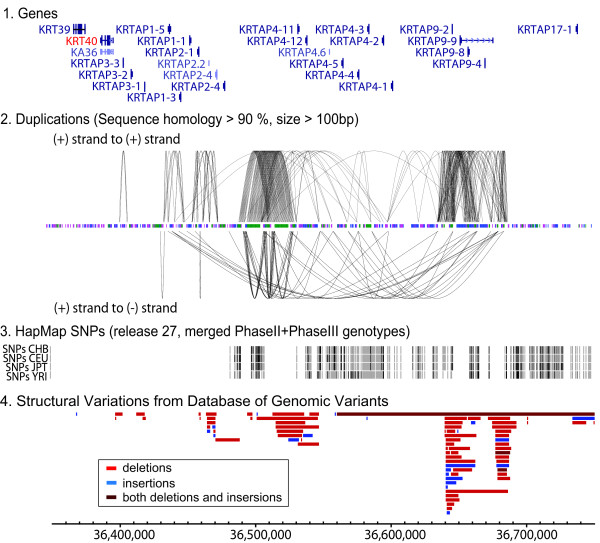
**A complex genomic region at a common copy-number breakpoint of the *ERBB2 *amplicon**. For the 400-kb region (from 36.350 to 36.750 Mb in hg18), duplicated segments (1), genes (2), the locations of HapMap SNPs in four major populations (Release 27) (3), and copy-number variants (from the Database of Genomic Variants) (4) are shown. In (1), duplicated segments are shown for either direct repeats (top) or inverted repeats (bottom). The distribution of repetitive sequences is also shown between the direct and inverted repeats. The (2), (3), and (4) were obtained from UCSC genome browser.

What is a unique property of the genomic region surrounding the *KRT40 *gene? Is the region fragile and prone to DNA rearrangements? To address these questions, we conducted an extensive characterization of the region. The region consists of a gene family of keratin-associated protein (KRTAP) genes; 21 *KRTAP *genes are within the region (Figure 4-1). The *KRTAP *genes encode a major component of hair in mammals and play an essential role in the formation of rigid and resistant hair shafts [73,74]. Such a large number of genes for a single gene family could be derived from gene duplications during genome evolution and would create complex genomic regions harboring segments of high sequence identities.

To determine the duplication contents, we scanned every 500-bp window in the region by Blat (UCSC genome Browser) and plotted segments that have more than 90% sequence homology (> 100-bp) with other windows (Figure 4-2 and Additional file, Table S1). We used a 100-bp cutoff rather than the conventional 1-kb cutoff, as such a short stretch of homology could still facilitate gene amplification [54,55]. A number of duplicated segments were identified within the region, both in the same strands (direct repeats, top) and between the complement strands (inverted repeats, bottom). Two large clusters of direct duplications are found (at around the coordinate 36.5-Mb and 36.65-Mb), and one of the duplications is 18-kb in size. These duplicated segments are not due to the extremely high content of repetitive elements, such as SINE elements, because the proportion of repetitive elements is very similar throughout the 3-Mb region surrounding the complex region (see Additional file, Figure S1).

Such extensive duplications create regions that are complex and difficult to investigate with current genomic approaches [75,76]. Failure to recognize duplications can lead to misinterpretation of marker genotypes [77,78]. For example, duplicated segments make it difficult to distinguish whether single-nucleotide changes are either the difference between duplicated segments (paralogous sequence variants) or allelic sequence variants (single-nucleotide polymorphisms, SNPs) [79,80]. Indeed, a set of SNPs that tag haploblocks in the human genome (HapMap SNPs, Release 27), an essential component of disease-association studies, is less well defined. An 110-kb region on the centromeric side does not have HapMap SNPs. Structural variants are common, and four deletion polymorphisms are within the region listed in the Database of Genomic Variants [81].

### Sequence divergence between duplicated segments

Previous studies showed the association between somatic breakpoints in cancer genomes and evolutionary breakpoints [82,83]. Because segmental duplications colocalize with evolutionary breakpoints in primate genomes [84,85], duplication activities during primate evolution could illustrate the unstable nature of a complex genomic region.

**Figure 5:**
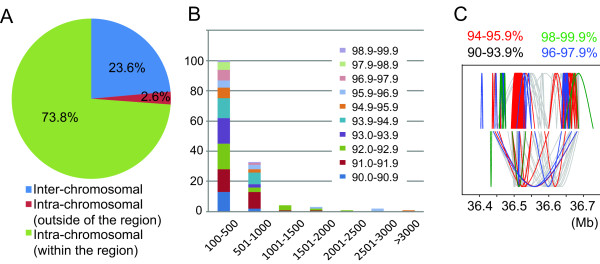
**Recurrent duplications of genomic segments within the complex region during primate evolution**. **(A) **A pie chart showing the proportion of duplications within the complex region, duplications between the complex region and outside of the region in the same chromosome, and duplications between the complex region and different chromosomes (interchromosomal duplications). Intrachromosomal duplications within the complex region account for three fourths of all the duplications. **(B) **Duplications within the complex region are binned based on size (x-axis), and the number of duplications for each bin is shown in the bar graph. A unique color is given based on the sequence identity between duplicated segments. **(C) **Inferred duplication activities within the complex region. Duplications are binned into four groups based on the sequence identity between duplicated segments, and older duplications (duplications with lower sequence identities) are overlaid by the newer duplications (duplications with higher sequence identities).

First, we determined the frequency of duplicated segments for (a) duplications within the complex region, (b) duplications between the complex region and other regions in the same chromosome, and (c) duplications between the complex region and other regions in different chromosomes (Figure 5A). Duplications occurred predominantly (73.6%) within the complex region, suggesting that the recombination between duplicated segments within the region may also be frequent in somatic cells.

The frequency of duplication events during evolution could in part be addressed by sequence divergence between duplications. When a segment was duplicated, the resulting two segments were 100% identical in their DNA sequences. Mutations could have accumulated on each segment, which results in sequence divergence between two segments (the proportion of sequences that differs between duplicated segments). Assuming that mutations accumulate in a neutral fashion, whether duplications are newer or older could be in part inferred by using sequence divergence [86].

When we group the duplicated segments based on the sequence identities, sequence identities vary for each duplicated pair (Figure 5B). A large number of small duplicated segments (less than 1 kb) exist in which sequence identities differ between segments, ranging from 90% to nearly 100%. This is also the case for larger duplicated segments; the largest segment (18 kb) has sequence identity of 95.6%, whereas most of the 1- to 2-kb segments have 92% to 93% sequence identity.

Although gene conversion homogenizes duplicated segments and limits our ability to date duplications precisely by using sequence divergence [87,88], these results indicate that duplications have possibly occurred many times within the complex region during primate genome evolution.

### Deletion polymorphisms within the complex region

Recombination between closely located repeats plays a critical role in the initiation of gene amplification in both mammalian cells and unicellular organisms [53-55,89]. We previously showed that as small as 79-bp DNA inverted repeats significantly increased the occurrence of gene amplification in mammalian cells [54]. Given the presence of duplicated segments and their structural variants within the region, a particular segment could promotes *ERBB2 *amplification, structural variants of which could be linked to the occurrence of *ERBB2 *amplification. Identifying such a segment directly might be difficult, however, because of the complexity of the region.

As an initial step, we defined haplotypes within the region. Different haplotypes could carry different genomic segments, and one haplotype could be associated with *ERBB2 *amplification. Because *ERBB2 *amplification occurs in 10% to 20% of breast tumors in all three major populations [90,91], the haplotype should likely be a common one in all populations. To define common haplotypes, we first searched for common deletion polymorphisms within the region from the Database of Genomic Variants (DGV) and the dbSNP database. Because of the paucity and the confounding effect from paralogous variants, SNP genotypes may not be as reliable as those of a deletion polymorphism. Furthermore, we could design a PCR-based genotyping assay for a deletion polymorphism to confirm that the variants are allelic, but not paralogous [92].

Although a number of studies reported deletion polymorphisms within the region, only two studies conducted genotyping on a population scale: copy-number variants studies from McCarroll *et al. *[57] for 270 HapMap samples and Conrad *et al. *[58] for 450 individuals of European, African, and East Asian ancestry: YRI (Yoruba in Ibadan, Nigeria), CEU (Utah residents with Northern and Western European ancestry from the CEPH collection), and CHB+JPT (Han Chinese in Beijing, China, and Japanese in Tokyo, Japan). Among the four (in MacCarroll *et al*.) and five (in Conrad *et al*.) deletion polymorphisms described in these studies within the region, only one is a common polymorphism (minor allele frequency > 5%). The polymorphism is located at the telomeric end of the complex region and overlaps with a 5.9-kb deletion polymorphism (rs72137527 from dbSNP database).

**Figure 6:**
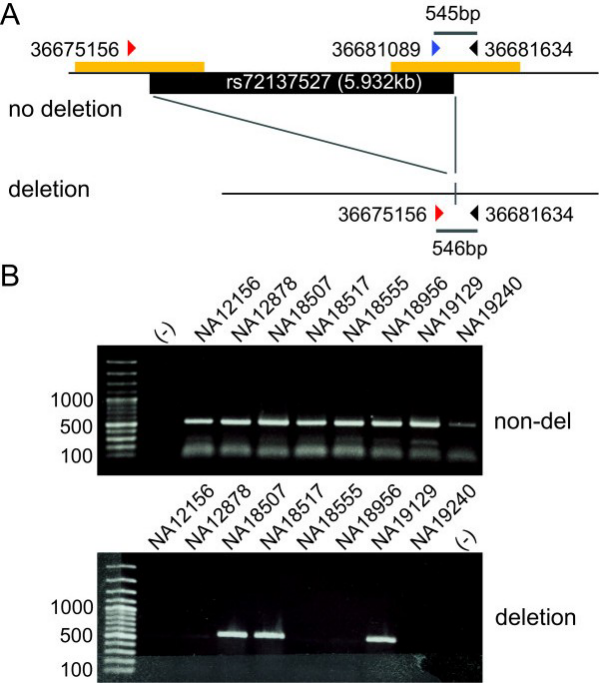
**Genotyping polymerase chain reaction (PCR) for the deletion polymorphism rs72137527**. **(A) **A PCR strategy for the deletion polymorphism located at the telomeric end of the complex region. Two independent forward primers (36675156 and 36681089) were paired with a common reverse primer (36681634) for amplifying either the nondeletion allele or the deletion allele. **(B) **Ethidium bromide staining gels are shown for either the PCR amplification of the deletion allele (deletion) or that of the nondeletion allele (non-del). DNA from 8 HapMap individuals was used.

To confirm that rs72137527 is the deletion polymorphism, we developed a genotyping PCR assay and genotyped several HapMap individuals (Figure 6). First, the genotypes from 10 HapMap trios (father, mother, and offspring) were consistent with the pattern of mendelian inheritance. Thus, the deletion was confirmed as an allelic polymorphism, not as paralogous variants. Second, the genotyping results by PCR assay were highly consistent (35 of 38 individuals) with the previous study [58], indicating that rs72137527 is the deletion polymorphism genotyped by two studies. The deletion was likely to have occurred by nonallelic homologous recombination (NAHR [93-96]), as it is flanked by 679-bp duplicated segments that are 92.1% similar to each other.

### Haploblocks within the complex genomic region

**Figure 7:**
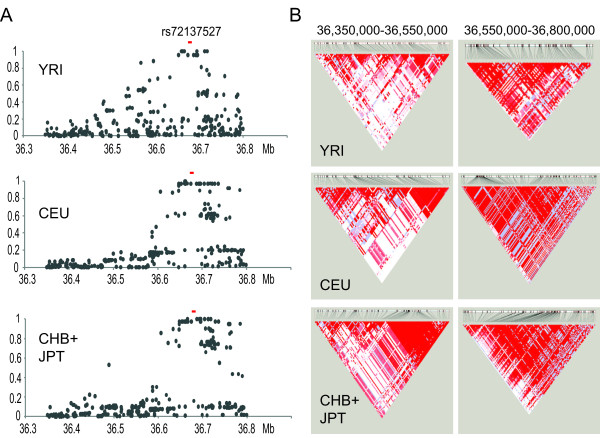
**Haploblocks within the complex genomic region (chr17: 36,350,000-36,750,000 in hg18)**. **(A) **Linkage disequilibrium between the deletion polymorphism (rs72137527) and HapMap single-nucleotide polymorphisms (SNPs). The *r^2 ^*values between the rs72137527 and each HapMap SNP are plotted against the physical locations of each SNP. **(B) **Haploblocks for the entire complex region. Triangular plots were generated by using Haploview for the HapMap SNP genotypes from three major populations (release 28) and are shown each for a centromeric half and a telomeric half. Red, strong linkage; white, no linkage.

Deletion polymorphisms and SNPs are very often in linkage disequilibrium (LD) [97,98]. The extent of a haplotype (haploblock) harboring the deletion polymorphism can be determined by the LD analysis between the deletion polymorphism and HapMap SNPs. To define the LD, we calculated the squared correlation coefficient *r*^2 ^between the deletion polymorphism and SNPs for three major populations (Figure 7 and see Additional file, Table S2). We found that several SNPs are in strong LD with the deletion polymorphism in all three populations. The LD blocks (*r^2 ^*> 0.9) extend a longer distance for CEU (27 SNPs, 114.48-kb) and CHB+JPT (31 SNPs, 137.72-kb) than YRI (17 SNPs, 65.17 kb) (see Additional file, Table S2). We also noticed that LD decreases gradually with distance for YRI. In contrast, LD is discontinuous for both CEU and CHB+JPT. The smaller LD block for African populations is consistent with the previous observations and may reflect a population bottleneck when modern humans first left Africa [99].

We then used Haploview to illustrate haploblocks for the entire region by using the SNP genotypes from the HapMap Release 28 (Figure 7B), the newer release that fills the 110-kb SNP gap (Figure 4) in Release 27. Consistent with the LD analysis between the deletion polymorphism and SNPs, a large haploblock is found for the telomeric side of the complex genomic region. However, a haploblock is less clear and smaller for the centromeric side of the complex region. Given the fact that the centromeric regions do not have as many duplicated segments as the telomeric region (Figure 4), having a large gap in the HapMap Release 27 seems unexplainable. The centromeric side may have unusual features and will require further characterization for identifying better genotyping markers.

## Discussion

In this study, we described a common copy-number breakpoint that potentially initiates *ERBB2 *amplification in primary breast tumors. The region is complex and consists of a large number of duplicated segments that form direct and inverted repeats. The sequence identities between duplicates are very high (> 90%), and some of them are more than 99% identical to each other. These duplicated segments are associated with the *KRTAP *gene family members, but not with high-copy repeats, such as SINE elements. Duplications appear to have occurred recurrently and predominantly within the region during primate evolution. These results suggest that the complex region could be more fragile than other unique loci and could play a mechanistic role in *ERBB2 *amplification.

Several lines of evidence support the unstable nature of complex genomic regions in the human genome. First, genomic regions with duplicated segments are preferred sites of non-disease-causing structural (copy number) variants [56,100]. The increased frequency of structural variants is due to the recombination between duplicated segments (non-allelic homologous recombination, NAHR [93-96]) in the germline. NAHR between segmental duplications leads to deletions, duplications, and inversions. Second, NAHR between duplicated segments also causes clinical phenotypes called genomic disorders [93-96,101]. NAHR between duplicated segments occurs recurrently and generates either duplications or deletions that determine the phenotypes of diseases. Recurrent NAHR for genomic disorders further supports the unstable nature of complex regions. Furthermore, the blocks of duplicated segments have been shown to be the most dynamic regions of the genome during primate evolution [102-104].

These facts would strongly argue for the unstable nature of complex genomic regions. Indeed, the important role of segmental duplications in creating somatic mutations in cancers is emerging. The breakpoints of isochromosome 17q, the most common isochromosome in human malignancy, was located within a large (> 30) inverted segmental duplication on 17p (isochromosome 17q, i17q) [105-107]. Translocation between chromosome 9 and 22, t(9;22)(q34;q11) causes the *BCR/ABL *gene fusion that is the underlying etiology of chronic myeloid leukemia (CML) [108]. From 10% to 20% of the translocation occurred between the 76-kb interchromosomal segmental duplications that are located either at the centromere proximal to *ABL *on chr 9 or the centromere distal to *BCR *on chr 22. The involvement of segmental duplications was also described for the microdeletion of *PTEN *tumor-suppressor gene in aggressive prostate cancers [109].

At the chromosome level, breakage-fusion-bridge (BFB) cycles are likely an underlying mechanism of *ERBB2 *amplification for at least a subset of breast tumors, as (a) the *ERBB2 *amplicons predominantly reside within a chromosome [61-65], and (b) copy-number loss at the telomeric side of the complex genomic regions (Figure 2) indicates chromosome breaks resulting in the loss of genetic materials. The BFB cycles have been shown to establish intrachromosomal amplicons for other oncogenes, such as *CCND1 *[110,111]. *CCDN1 *resides at chromosome 11q13 and is frequently amplified in head and neck tumors. *CCND1 *is surrounded by three clusters of segmental duplications. These clusters have been shown to colocalize with the boundaries of amplified regions [112], suggesting that a series of rearrangements could occur within these clusters during BFB cycles. In this regard, it is noteworthy that, in addition to the complex region described in this study, additional complex regions exist within *ERBB2 *amplicons. At the centromeric side, two large (a few hundred kb) euchromatic gaps of human genome assembly (hg18) are noted: one at 1.5 mega-base (Mb) and another at 3.3 Mb centromeric side of *ERBB2 *(see Additional file 1, Figure S2) [113]. Assembly gaps represent regions with full duplicated DNAs and/or complex, unclonable regions. Similar to *CCND1 *amplicon, these duplicated DNAs within gaps may serve as substrates for DNA rearrangements during BFB cycles.

We further found that other commonly amplified genes are also in close proximity to complex genomic regions. Among the 13 cancer genes that are most commonly amplified and overexpressed [114], five genes (*ERBB2*, *CCND1*, *MYCL1*, *MDM4*, and *MYCN*) are located within 1.5 Mb from either assembly gaps or blocks of duplicated segments (see Additional file, Table S3). Additionally, chromosome 1q21, a commonly amplified region in many tumor types, has 18 gaps within 6 Mb. In contrast, neither complex genomic regions nor assembly gaps are seen within the 6-Mb region surrounding *MYC *oncogene, which could explain a different mechanism for *MYC *amplification [115].

At the DNA level, sequence homology between duplicated segments could play an initiating role in BFB cycles and gene amplification. By using model systems, we and others showed that inverted repeats preexisting in the genome can nucleate the duplication of large genomic segments [22,53-55,89]. Duplicated segments could facilitate the initiation of BFB cycles in two ways.

First, inverted repeats can adopt Holliday junction-like structure by forming a cruciform. The resolution of a cruciform results in two chromosomal parts with hairpin-capped ends. The replication of a centromere-harboring part with a hairpin-capped end results in the formation of a dicentric chromosome and the initiation of BFB cycles.

**Figure 8:**
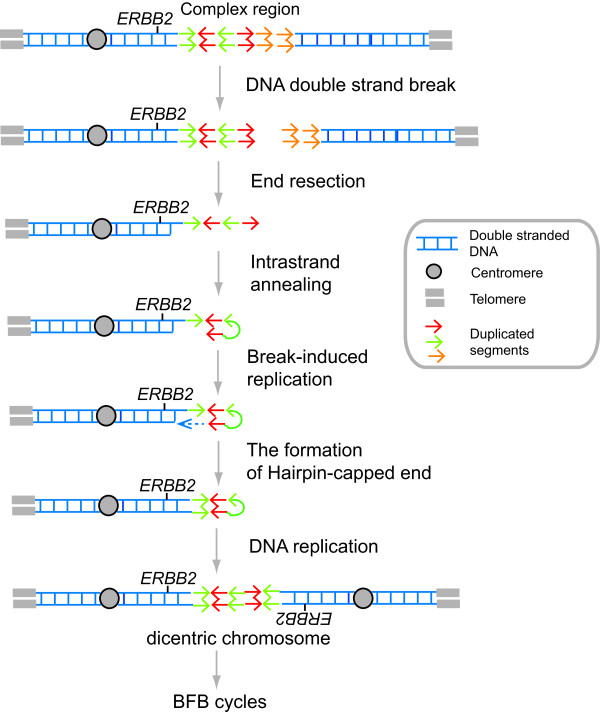
**Nonallelic homologous recombination (NAHR) between duplicated segments initiates the BFB cycles (model)**. Duplicated segments within a complex region could adopt a complex secondary structure that can impose an obstacle for the progression of replication forks and would generate a DSB. The 5'- to 3'-end resection of a DSB exposes single-stranded DNA that would fold back and anneal between inverted repeat sequences (intrastrand annealing). BIR (break-induced replication) would prime DNA synthesis and fill the single-stranded gap to create a chromosome with a hairpin-capped end. The replication of the chromosome would generate a dicentric chromosome that initiates the BFB cycles.

Second, duplicated segments could adopt a complex secondary structure that can impose an obstacle to the progression of replication forks (Figure 8) [116,117]. As replication fork stalling and collapse could be processed into one-ended DNA breaks [118], the complex regions may have increased DNA breaks. The 5*'*- to 3*'*-end resection of one-ended DNA breaks exposes single-stranded DNA [119]. When the end of single-stranded DNA folds back and anneals to an inverted repeat sequence (intrastrand annealing [55]), it would prime DNA synthesis (break-induced replication, BIR [120]) and fill in the single-stranded gap to create a chromosome with a hairpin-capped end. Thus, the sequence homology between duplicated segments could be mutagenic and initiate BFB cycles.

In this regard, it is noteworthy that *ERBB2 *amplification is absent in breast tumors from *BRCA1 *mutation carriers [121]. *BRCA1 *binds to many proteins of DNA damage response and repair and thus plays a critical role in maintaining genome integrity [122]. *BRCA1 *is recruited to the chromatin with damaged DNA very early [123,124] and stimulates DNA end resection for homology-directed repair [125,126]. As *BRCA1 *mutant cells could lack efficient end resection, both mutation-free (conservative) and mutagenic homology-directed repair pathways could be impaired [127]. The conservative pathway is RAD51 dependent and repairs DSBs by using sister chromatids as a template, whereas the mutagenic pathway can be RAD51 independent [128] and could use repeated segments as a template. Therefore, the fact that *ERBB2 *amplification is rare in tumors with *BRCA1 *mutation may indicate that *ERBB2 *amplification is dependent on mutagenic homology-directed repair. In contrast, 15% of tumors derived from *BRCA2 *mutation carriers have *ERBB2 *amplification [121]. *BRCA2 *also functions for homology-directed repair; however, it has a more-specific role. *BRCA2 *has a RAD51-binding domain and plays an important role in conservative repair [129,130]. Indeed, in *BRCA2 *mutant cells, conservative repair was impaired, but mutagenic repair was not affected [127]. Therefore, the distinct *ERBB2 *amplification tendency between *BRCA1 *and *BRCA2 *mutant careers further suggests the involvement of recombination between repeated segments in *ERBB2 *amplification.

Alternatively, BIR initiated from one-ended DNA breaks at the sites of collapsed replication forks could be more processive, and repeated template switching (fork stalling and template switching, FoSTeS [131]) could result in complex genomic rearrangements and copy-number transition [132,133]. Newly established forks from one-ended DNA breaks could invade into either sister chromatid or homologues at nonallelic loci by using duplicated sequences or microhomology [134,135]. Invading strands can be unstable and often dissociate from template strands. The resulting free ends would repeat invasion several times at nonallelic loci to create complex genomic rearrangements. Copy-number increases from such complex rearrangements is relatively low, from twofold to threefold [132]. However, duplication and triplication of the segments could facilitate further rearrangements (for example, unequal sister chromatid exchange) and high-level amplification.

*ERBB2 *amplicons have been classified into two groups: a large amplicon including the *TOP2A *gene and a smaller, more-restricted amplicon (without *TOP2A*) surrounding the *ERBB2 *gene [66,67]. *TOP2A *encodes a DNA topoisomerase II (topoII) that controls and alters the topologic state of DNA in several aspects of DNA metabolism, such as chromosome segregation, transcription, and chromatin organization [136-138]. Because the complex region is located at the telomeric side of *TOP2A *gene, tumors having a breakpoint at the region belong to the *TOP2A*-coamplified tumors. Whether tumors without *TOP2A *amplification have independent common copy-number breakpoints is an important issue for future studies. It is also possible that an initiating break/recombination occurs at the complex region (or on a further telomeric side [139]) and, during the evolution of the amplicon, secondary rearrangements could delete both the region including *TOP2A *and the complex region from the amplicon. *TOP2A *deletion in *ERBB2*-amplified tumors is common [66,140]. Even in coamplified tumors, *TOP2A *and *ERBB2 *resided in different chromosomal domains [64,141], suggesting that secondary rearrangements separated the two genes from primary amplicons.

Given the established role of repeated segments in gene amplification in experimental systems, structural variants of such segments could have a significant effect in the occurrence of *ERBB2 *amplification [22]. Several structural variants are reported within the region, and some of them could be good candidates for the variants. However, defining the DNA sequences at breakpoints and identifying the segments responsible for *ERBB2 *amplification can be hampered by the complexity of the region. Therefore, as an initial step, we made an effort to define the haploblocks within the region. By combining the genotyping data from the deletion polymorphism and SNP genotypes, we were able to define two blocks, one of which showed a strong LD within the block. Our ongoing effort for further defining haploblocks and identifying genetic markers will provide a better understanding of the complex region. Such genetic markers could be useful, especially for the genomic regions where SNP markers are less well defined and genome-wide association studies [142,143] may have a limited power.

## Conclusions

We show here a potential initiating role of a complex genomic region in *ERBB2 *amplification in breast cancer. The genomic sequence of the region is still ambiguous, as Genome Reference Consortium is providing an alternative sequence assembly for the region. Furthermore, two large sequence gaps (in hg18) exist on the centromeric side of *ERBB2 *(see Additional file, Figure S2). These sequence gaps likely contain many repeated sequences and structural variants and could also be fragile. Therefore, *ERBB2 *is flanked by many complex genomic regions that may not be sufficiently investigated by current genomic technologies. Investigating such regions in detail, including the patterns of DNA rearrangements at the nucleotide level, structural variants, and haplotypes within the regions, is important for the mechanistic study of *ERBB2 *amplification.

## Abbreviations

Array-CGH: array-comparative genomic hybridization; BFB cycles: breakage-fusion-bridge cycles; *CCND1*: cyclin D1 gene; *ERBB2*: v-erb-b2 erythroblastic leukemia viral oncogene homolog 2; FISH: fluorescence *in situ *hybridization; HER2: human epidermal growth factor receptor 2; *KRT40*: keratin 40 gene; *KRTAP*: keratin-associated protein gene; IHC: immunohistochemistry; NAHR: nonallelic homologous recombination; *TOP2A*: topoisomerase 2A gene.

## Competing interests

The author(s) declare that they have no competing interests.

## Authors' contributions

MM and HT wrote the manuscript. MM and AI carried out the molecular genetic experiments. MM conducted SNP analyses. AK and RT conceived of the study, participated in its design and coordination, and helped to draft the manuscript. XC and RS conducted the microarray data analyses. GTB, JC, JL, and RT provided breast tumor tissues. All authors read and approved the final manuscript.

## Supplementary Material

Additional file 1 **Figure S1**. Repeat Masker Chr17 35-38 Mb (p.2). **Figure S2**. Sequence gaps near *ERBB2 *(in hg18) (p. 3). **Table S1**. Chromosome 17 BLAT results (p. 4 to 24). **Table S2**. LD map boundaries (p. 25). **Table S3**. Cancer gene amplification and complex genomic regions (p. 26). **Table S4**. HapMap sample ID list (p. 27). **Table S5**. PCR and qPCR primer list (p. 28). Click here for file
