# Supplementary material for: A common copy-number breakpoint of ERBB2 amplification in breast cancer colocalizes with a complex block of segmental duplications
Source: Breast Cancer Res. 2012 Nov 26;14(6):R150. doi: 10.1186/bcr3362 (PMC4053137; doi:10.1186/bcr3362)
Supplement: Additional file 1 — Figure S1. Repeat Masker Chr17 35-38 Mb (p.2). Figure S2. Sequence gaps near ERBB2 (in hg18) (p. 3). Table S1. Chromosome 17 BLAT results (p. 4 to 24). Table S2. LD map boundaries (p. 25). Table S3. Cancer gene amplification and complex genomic regions (p. 26). Table S4. HapMap sample ID list (p. 27). Table S5. PCR and qPCR primer list (p. 28). [file bcr3362-S1.PDF]

Additional File 1:

Table of Contents:

I. Figure S1. Repeat Masker Chr17 35-38Mb (p.2)

II. Figure S2. Sequence gaps near *ERBB2* (in hg18) (p. 3)

III. Table S1. Chromosome 17 BLAT results (p. 4-24)

IV. Table S2. LD Map Boundaries (p. 25)

V. Table S3. Cancer Gene amplification and complex genomic regions (p. 26)

VI. Table S4. HapMap Sample ID List (p. 27)

VII. Table S5. PCR and qPCR Primer List (p. 28)

Figure S1. Repeat Masker Chr17 35-38Mb

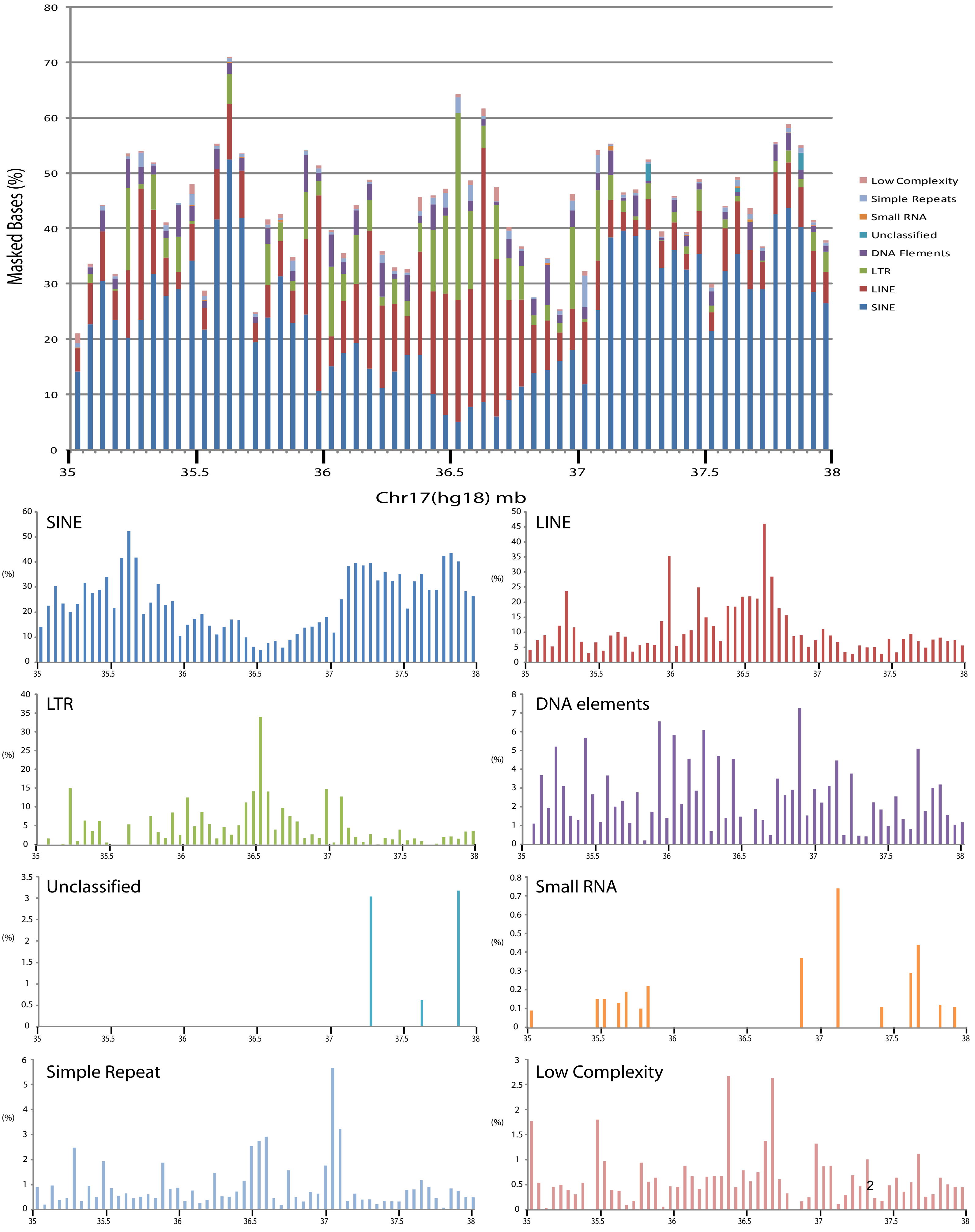

Figure S2. Sequence gaps near *ERBB2* (in hg18)

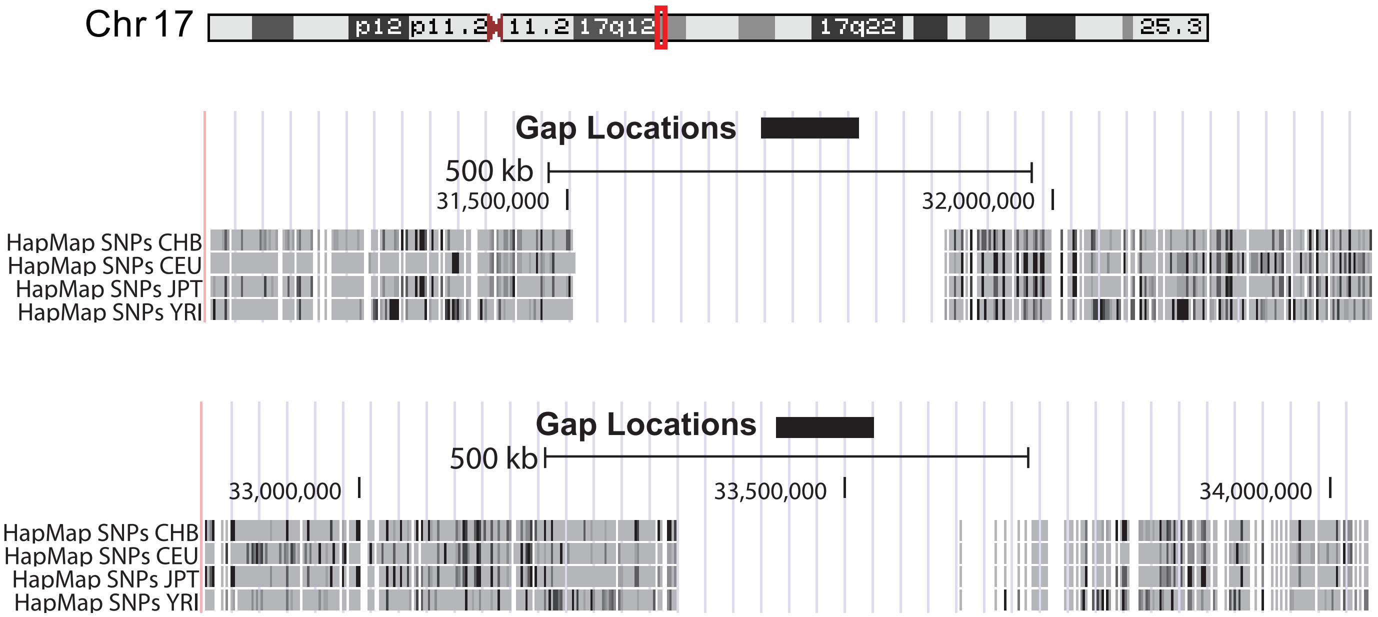

Table S1. Chr17 BLAT Results

The worksheet contains 500 bp increments of DNA sequence from hg18 that were BLAT to the hg18 genome
Similarity Criteria: >90%, >100bp

Column	ID	Description
A	Start	Start of 500 bp segment to be run through UCSC Genome BLAT
B	End	End of 500 bp segment to be run through UCSC Genome BLAT
C	BLAT Result	BLAT query result (yes = positive hit of >90% similarity, >100 bp to within 400 kb region of interest, nothing = no hits, many inter/intra = many hits of >90% similarity, >100 bp throughout the genome
D	start	Location within 500 bp segment that BLAT similarity begins. This value will be between 1-500 since the segment used to perform the BLAT was a 500 bp segment.
E	end	Location within 500 bp segment that BLAT similarity ends. This value will be between 1-500 since the segment used to perform the BLAT was a 500 bp segment.
F	Qsize	Query sequence size
G	% alignment	% alignment to similar sequence
H	chr	The chromosome where the similar sequence is
I	strand	The orientation to which the similarity is located
J	start	The start of similarity to the 500 bp fragment (mapped to a particular region using the hg18 genome as reference)
K	end	The end of similarity to the 500 bp fragment (mapped to a particular region using the hg18 genome as reference)
L	span	The total span of similarity. Note that in some cases this span is greater than the 500 bp.

Start	End	BLAT Result	start	end	Qsize	% alignment	chr	strand	start	end	span
36,350,000	36,350,500	nothing									
36,350,500	36,351,000	nothing									
36,351,000	36,351,500	many inter/intra									
36,351,500	36,352,000	many inter/intra									
36,352,000	36,352,500	many inter/intra									
36,352,500	36,353,000	many inter/intra									
36,353,000	36,353,500	many inter/intra									
36,353,500	36,354,000	many inter/intra									
36,354,000	36,354,500	nothing									
36,354,500	36,355,000	nothing									
36,355,000	36,355,500	many inter/intra									
36,355,500	36,356,000	nothing									
36,356,000	36,356,500	nothing									
36,356,500	36,357,000	nothing									
36,357,000	36,357,500	nothing									
36,357,500	36,358,000	nothing									
36,358,000	36,358,500	many inter/intra									
36,358,500	36,359,000	many inter/intra									
36,359,000	36,359,500	many inter/intra									
36,359,500	36,360,000	many inter/intra									
36,360,000	36,360,500	nothing									
36,360,500	36,361,000	many inter/intra									
36,361,000	36,361,500	nothing									
36,361,500	36,362,000	nothing									
36,362,000	36,362,500	nothing									
36,362,500	36,363,000	yes	159	283	500	92.8	17	-	19553352	19553477	126
		yes	78	223	500	95.7	10	-	120638480	120638986	507
36,363,000	36,363,500	nothing									
36,363,500	36,364,000	nothing									
36,364,000	36,364,500	nothing									
36,364,500	36,365,000	nothing									
36,365,000	36,365,500	nothing									
36,365,500	36,366,000	nothing									
36,366,000	36,366,500	nothing									
36,366,500	36,367,000	nothing									
36,367,000	36,367,500	yes	37	155	500	90.2	1	-	154248731	154249156	426
36,367,500	36,368,000	nothing									
36,368,000	36,368,500	nothing									
36,368,500	36,369,000	nothing									
36,369,000	36,369,500	nothing									
36,369,500	36,370,000	nothing									
36,370,000	36,370,500	nothing									
36,370,500	36,371,000	many inter/intra									
36,371,000	36,371,500	nothing									
36,371,500	36,372,000	nothing									
36,372,000	36,372,500	nothing									
36,372,500	36,373,000	many inter/intra									
36,373,000	36,373,500	many inter/intra									
36,373,500	36,374,000	nothing									
36,374,000	36,374,500	nothing									
36,374,500	36,375,000	many inter/intra									
36,375,000	36,375,500	many inter/intra									
36,375,500	36,376,000	many inter/intra									

36,376,000	36,376,500	yes	166	352	500	97.8	17	+	36779179	36850392	71214
36,376,500	36,377,000	nothing									
36,377,000	36,377,500	nothing									
36,377,500	36,378,000	many inter/intra									
36,378,000	36,378,500	many inter/intra									
36,378,500	36,379,000	many inter/intra									
36,379,000	36,379,500	nothing									
36,379,500	36,380,000	many inter/intra									
36,380,000	36,380,500	many inter/intra									
36,380,500	36,381,000	many inter/intra									
36,381,000	36,381,500	many inter/intra									
36,381,500	36,382,000	yes	68	190	500	95.4	13	+	85348192	85379517	31326
36,382,000	36,382,500	nothing									
36,382,500	36,383,000	many inter/intra									
36,383,000	36,383,500	many inter/intra									
36,383,500	36,384,000	many inter/intra									
36,384,000	36,384,500	many inter/intra									
36,384,500	36,385,000	many inter/intra									
36,385,000	36,385,500	many inter/intra									
36,385,500	36,386,000	many inter/intra									
36,386,000	36,386,500	many inter/intra									
36,386,500	36,387,000	yes	257	500	500	91.3	17	-	20452920	20453164	245
		yes	298	494	500	96.1	1	-	36050015	36050423	409
36,387,000	36,387,500	many inter/intra									
36,387,500	36,388,000	nothing									
36,388,000	36,388,500	nothing									
36,388,500	36,389,000	nothing									
36,389,000	36,389,500	many inter/intra									
36,389,500	36,390,000	many inter/intra									
36,390,000	36,390,500	nothing									
36,390,500	36,391,000	nothing									
36,391,000	36,391,500	nothing									
36,391,500	36,392,000	many inter/intra									
36,392,000	36,392,500	nothing									
36,392,500	36,393,000	many inter/intra									
36,393,000	36,393,500	yes	115	232	500	94.8	18	+	56354429	56354547	119
36,393,500	36,394,000	nothing									
36,394,000	36,394,500	nothing									
36,394,500	36,395,000	nothing									
36,395,000	36,395,500	nothing									
36,395,500	36,396,000	yes	215	500	500	89.6	1	-	162018355	162018638	284
36,396,000	36,396,500	many inter/intra									
36,396,500	36,397,000	nothing									
36,397,000	36,397,500	nothing									
36,397,500	36,398,000	many inter/intra									
36,398,000	36,398,500	many inter/intra									
36,398,500	36,399,000	many inter/intra									
36,399,000	36,399,500	many inter/intra									
36,399,500	36,400,000	many inter/intra									
36,400,000	36,400,500	nothing									
36,400,500	36,401,000	many inter/intra									
36,401,000	36,401,500	many inter/intra									
36,401,500	36,402,000	nothing									
36,402,000	36,402,500	nothing									
36,402,500	36,403,000	nothing									
36,403,000	36,403,500	nothing									
36,403,500	36,404,000	yes				97.90%	17	+	36409311	36409634	323
36,404,000	36,404,500	nothing									
36,404,500	36,405,000	nothing									
36,405,000	36,405,500	nothing									
36,405,500	36,406,000	nothing									
36,406,000	36,406,500	nothing									
36,406,500	36,407,000	nothing									
36,407,000	36,407,500	yes	27	193	500	92.4	13	+	63645445	63645931	487
36,407,500	36,408,000	nothing									
36,408,000	36,408,500	nothing									
36,408,500	36,409,000	nothing									
36,409,000	36,409,500	yes	312	500	500	99.00%	17	+	36403555	36403903	349
36,409,500	36,410,000	yes	1	135	500	97.1	17	+	36403744	36403878	135
36,410,000	36,410,500	nothing									
36,410,500	36,411,000	nothing									
36,411,000	36,411,500	many inter/intra									
36,411,500	36,412,000	nothing									
36,412,000	36,412,500	many inter/intra									
36,412,500	36,413,000	nothing									
36,413,000	36,413,500	many inter/intra									
36,413,500	36,414,000	many inter/intra									

36,414,000	36,414,500	yes				93.4	17	+	36418696	36418771	76
36,414,500	36,415,000	nothing									
36,415,000	36,415,500	nothing									
36,415,500	36,416,000	many inter/intra									
36,416,000	36,416,500	nothing									
36,416,500	36,417,000	many inter/intra									
36,417,000	36,417,500	yes				92.5	17	-	34044333	34044439	107
		many inter/intra									
36,417,500	36,418,000	nothing									
36,418,000	36,418,500	nothing									
36,418,500	36,419,000	yes				97.5	17	+	36403763	36403801	39
36,419,000	36,419,500	nothing									
36,419,500	36,420,000	many inter/intra									
36,420,000	36,420,500	many inter/intra									
36,420,500	36,421,000	nothing									
36,421,000	36,421,500	nothing									
36,421,500	36,422,000	nothing									
36,422,000	36,422,500	nothing									
36,422,500	36,423,000	yes	51	460	500	91.2	1	+	75177265	75177722	458
36,423,000	36,423,500	nothing									
36,423,500	36,424,000	nothing									
36,424,000	36,424,500	nothing									
36,424,500	36,425,000	nothing									
36,425,000	36,425,500	nothing									
36,425,500	36,426,000	yes				91	10	+	128421041	128421469	429
36,426,000	36,426,500	many inter/intra									
36,426,500	36,427,000	many inter/intra									
36,427,000	36,427,500	yes				100	11	+	132016964	132017389	426
36,427,500	36,428,000	nothing									
36,428,000	36,428,500	nothing									
36,428,500	36,429,000	many inter/intra									
36,429,000	36,429,500	many inter/intra									
36,429,500	36,430,000	many inter/intra									
36,430,000	36,430,500	yes				91.3	11	+	129006443	129006706	264
36,430,500	36,431,000	yes				94.9	16	+	48727899	48728004	106
36,431,000	36,431,500	yes				98.8	17	-	36434315	36434472	158
		yes				93.7	7	+	63332186	63332516	331
		yes				94.6	18	+	40070990	40071173	184
36,431,500	36,432,000	many inter/intra									
		yes									
36,432,000	36,432,500	many inter/intra									
36,432,500	36,433,000	many inter/intra									
36,433,000	36,433,500	nothing									
36,433,500	36,434,000	nothing									
36,434,000	36,434,500	yes				98.8	17	-	36431072	36431229	158
36,434,500	36,435,000	nothing									
36,435,000	36,435,500	yes				100	16	-	3480340	3480798	459
		yes				100	4	+	37720965	37721352	388
		yes				93.8	4	+	39334296	39334622	327
36,435,500	36,436,000	many inter/intra									
36,436,000	36,436,500	yes				94.7	17	+	36444074	36444194	121
		yes				94.2	17	+	36439478	36439589	112
		yes				94.6	17	+	36450640	36450740	101
36,436,500	36,437,000	yes				91.3	17	+	36439591	36444656	5066
		yes				93	17	+	36444196	36451223	7028
		yes				92	17	+	36450742	36594641	143900
		yes				91.4	17	-	36659491	36685792	26302
		yes				93.5	17	-	36515157	36642757	127601
		yes				91.4	17	-	36659606	36665305	5700
		yes				91.2	17	-	36685468	36685702	235
36,437,000	36,437,500	nothing									
36,437,500	36,438,000	nothing									
36,438,000	36,438,500	many inter/intra									
36,438,500	36,439,000	yes				97.3	18	+	21655925	21656172	248
		yes				93.6	12	+	4825117	4825331	215
36,439,000	36,439,500	nothing									
36,439,500	36,440,000	yes				94.9	17	+	36450651	36451189	539
		yes				93.3	17	+	36444105	36596922	152818
		yes				91.4	17	+	36436418	36436956	539
		yes				93.1	17	-	36681080	36685909	4830
		yes				91.4	17	-	36636565	36636881	317
		yes				93.8	17	-	36659606	36659893	288
		yes				96.8	17	-	36681221	36681516	296
		yes				93.9	11	+	1599372	1599768	397
36,440,000	36,440,500	nothing									
36,440,500	36,441,000	yes				96.3	7	-	36010722	36011093	372
		yes				94.4	5	-	83657875	83658142	268

		yes				94	3	-	24003128	24003485	358
36,441,000	36,441,500	many inter/intra									
36,441,500	36,442,000	many inter/intra									
36,442,000	36,442,500	many inter/intra									
36,442,500	36,443,000	many inter/intra									
36,443,000	36,443,500	many inter/intra									
36,443,500	36,444,000	nothing									
36,444,000	36,444,500	yes				96	17	+	36439475	36451045	11571
		yes				94.7	17	+	36436387	36436833	447
		yes				90.3	17	-	36665258	36681529	16272
		yes				93.1	17	+	36577496	36577869	374
		yes				92.2	17	-	36659608	36681416	21809
36,444,500	36,445,000	yes				95.2	17	+	36439757	36594476	154720
		yes				94.7	17	-	36681281	36681548	268
36,445,000	36,445,500	many inter/intra									
36,445,500	36,446,000	many inter/intra									
36,446,000	36,446,500	nothing									
36,446,500	36,447,000	nothing									
36,447,000	36,447,500	nothing									
36,447,500	36,448,000	yes				92	12	+	120292205	120292357	153
36,448,000	36,448,500	many inter/intra									
36,448,500	36,449,000	many inter/intra									
36,449,000	36,449,500	many inter/intra									
36,449,500	36,450,000	many inter/intra									
36,450,000	36,450,500	nothing									
36,450,500	36,451,000	yes				97.5	17	+	36444103	36444422	320
		yes				93.6	17	+	36436407	36436788	382
		yes				94.8	17	+	36439498	36439820	323
		yes				96.8	17	-	36665281	36665381	101
36,451,000	36,451,500	yes				93.7	17	+	36444362	36444665	304
		yes				91.9	17	+	36436696	36436981	286
		yes				93.3	17	+	36594461	36594640	180
36,451,500	36,452,000	nothing									
36,452,000	36,452,500	nothing									
36,452,500	36,453,000	nothing									
36,453,000	36,453,500	nothing									
36,453,500	36,454,000	many inter/intra									
36,454,000	36,454,500	many inter/intra									
36,454,500	36,455,000	many inter/intra									
36,455,000	36,455,500	many inter/intra									
36,455,500	36,456,000	many inter/intra									
36,456,000	36,456,500	many inter/intra									
36,456,500	36,457,000	yes				100	17	+	36464524	36469782	5259
		yes				100	17	+	36475236	36475577	342
		yes				100	17	+	36464674	36464943	270
		yes				95.1	17	-	36622698	36642470	19773
		yes				95.6	17	-	36599841	36600072	232
		yes				97.3	17	-	36636849	36642470	5622
36,457,000	36,457,500	yes				95.8	17	+	36475578	36475742	165
		yes				99.4	17	+	36464944	36465090	147
		yes				96.5	17	+	36469783	36469922	140
		yes				94.6	16	+	49363159	49363661	503
36,457,500	36,458,000	many inter/intra									
36,458,000	36,458,500	nothing									
36,458,500	36,459,000	yes				93.1	17	+	36460632	36461294	663
		yes				90.7	17	+	36460569	36460703	135
		many inter/intra									
36,459,000	36,459,500	many inter/intra									
36,459,500	36,460,000	many inter/intra									
36,460,000	36,460,500	yes				94.3	17	+	36460863	36461280	418
		yes				90	17	-	36460768	36461334	567
		yes				92.1	17	-	36460798	36461257	460
		yes				90.5	17	-	36460826	36461312	487
		yes				98.5	17	+	36460924	36461188	265
		yes				92.2	17	-	36460682	36461290	609
		yes				95	17	+	36461029	36461188	160
		many inter/intra									
36,460,500	36,461,000	yes				95.1	17	+	36460688	36461034	347
		yes				90.4	17	-	36460769	36461252	484
		yes				94	17	-	36460562	36461343	782
		yes				97.4	17	+	36460742	36460950	209
		yes				93.9	17	-	36460738	36461201	464
		yes				92.9	17	+	36460669	36460869	201
		yes				96.4	17	-	36460669	36460983	315
		yes				94.7	17	-	36460816	36461209	394
		yes				96.5	17	-	36460711	36461090	380
		many inter/intra									

36,461,000	36,461,500	many inter/intra									
36,461,500	36,462,000	many inter/intra									
36,462,000	36,462,500	many inter/intra									
36,462,500	36,463,000	many inter/intra									
36,463,000	36,463,500	many inter/intra									
36,463,500	36,464,000	nothing									
36,464,000	36,464,500	nothing									
36,464,500	36,465,000	yes				100%	17	+	36456565	36457055	491
		yes				96.20%	17	+	36475236	36475633	398
		yes				96.20%	17	+	36469441	36469838	398
		yes				94%	17	-	36515596	36600072	84477
36,465,000	36,465,500	yes				91.50%	17	+	36469839	36469967	129
		yes				97.20%	17	+	36475634	36475740	107
36,465,500	36,466,000	nothing									
36,466,000	36,466,500	nothing									
36,466,500	36,467,000	yes	1	102	500	93.30%	14	+	84883010	84883310	301
36,467,000	36,467,500	nothing									
36,467,500	36,468,000	many inter/intra									
36,468,000	36,468,500	many inter/intra									
36,468,500	36,469,000	nothing									
36,469,000	36,469,500	nothing									
36,469,500	36,470,000	yes	6	468	500	97.50%	17	+	36464666	36465128	463
		yes	1	446	500	98.30%	17	+	36475295	36475740	446
		yes	1	423	500	98.90%	17	+	36456717	36457139	423
		yes	28	277	500	95.80%	17	-	36665564	36675361	9798
		yes	20	276	500	95.30%	17	-	36648201	36665364	17164
		yes	28	276	500	97.30%	17	-	36636849	36642470	5622
36,470,000	36,470,500	nothing									
36,470,500	36,471,000	yes	4	442	500	97.90%	10	+	8221735	8238024	16290
36,471,000	36,471,500	nothing									
36,471,500	36,472,000	nothing									
36,472,000	36,472,500	many inter/intra									
36,472,500	36,473,000	nothing									
36,473,000	36,473,500	nothing									
36,473,500	36,474,000	nothing									
36,474,000	36,474,500	nothing									
36,474,500	36,475,000	nothing									
36,475,000	36,475,500	yes	209	500	500	99.30%	17	+	36464444	36469704	5261
		yes	237	500	500	100.00%	17	+	36456658	36456921	264
		yes	237	500	500	96.40%	17	+	36464617	36464865	249
		yes	64	180	500	97.20%	12	-	27132326	27139118	6793
36,475,500	36,476,000	yes	1	310	500	93.30%	17	+	36456922	36457213	292
		yes	1	241	500	98.40%	17	+	36464866	36465106	241
		yes	1	241	500	96.70%	17	+	36469705	36469945	241
36,476,000	36,476,500	nothing									
36,476,500	36,477,000	nothing									
36,477,000	36,477,500	many inter/intra									
36,477,500	36,478,000	nothing									
36,478,000	36,478,500	nothing									
36,478,500	36,479,000	nothing									
36,479,000	36,479,500	nothing									
36,479,500	36,480,000	nothing									
36,480,000	36,480,500	nothing									
36,480,500	36,481,000	many inter/intra									
36,481,000	36,481,500	many inter/intra									
36,481,500	36,482,000	yes	216	431	500	90.80%	17	-	36559150	36559554	405
		yes	216	429	500	90.20%	17	-	36511642	36570478	58837
		yes	216	428	500	90.00%	17	-	36507578	36587951	80374
		yes	303	427	500	91.60%	17	-	36550012	36596833	46822
36,482,000	36,482,500	nothing									
36,482,500	36,483,000	many inter/intra									
36,483,000	36,483,500	many inter/intra									
36,483,500	36,484,000	yes	151	322	500	95.20%	19	-	23723406	23723670	265
36,484,000	36,484,500	many inter/intra									
36,484,500	36,485,000	many inter/intra									
36,485,000	36,485,500	nothing									
36,485,500	36,486,000	nothing									
36,486,000	36,486,500	nothing									
36,486,500	36,487,000	many inter/intra									
36,487,000	36,487,500	many inter/intra									
36,487,500	36,488,000	many inter/intra									
36,488,000	36,488,500	many inter/intra									
36,488,500	36,489,000	nothing									
36,489,000	36,489,500	nothing									
36,489,500	36,490,000	many inter/intra									
36,490,000	36,490,500	many inter/intra									
36,490,500	36,491,000	many inter/intra									

36,491,000	36,491,500	yes	1	500	500	97.00%	17	+	36512171	36512670	500
		yes	26	291	500	92.70%	5	-	84113751	84114047	297
36,491,500	36,492,000	yes	1	500	500	98.20%	17	+	36512671	36513176	506
		many inter/intra									
36,492,000	36,492,500	yes	1	500	500	93.80%	17	+	36513177	36513680	504
		yes	175	500	500	90.20%	17	-	36509661	36509986	326
36,492,500	36,493,000	yes	11	469	500	95.00%	17	+	36513691	36514149	459
		yes	1	500	500	91.40%	17	-	36509157	36509660	504
		yes	1	500	500	90.80%	17	-	36529371	36529877	507
36,493,000	36,493,500	yes	1	500	500	98.80%	17	+	36514183	36514681	499
		yes	1	259	500	91.10%	17	-	36508899	36509156	258
36,493,500	36,494,000	yes	1	500	500	98.80%	17	+	36514682	36515181	500
		yes	1	500	500	98.80%	17	+	36514682	36515181	500
		yes	1	500	500	91.20%	17	-	36507848	36528581	20734
		yes	146	500	500	91.70%	17	-	36528079	36528434	356
		yes	1	122	500	92.80%	17	-	36508230	36508351	122
		yes	258	500	500	94.50%	17	-	36533886	36534137	252
36,494,000	36,494,500	yes	1	500	500	97.20%	17	+	36515182	36515846	665
		yes	1	500	500	94.60%	17	-	36507258	36507847	590
		yes	1	500	500	93.40%	17	-	36527459	36594617	67159
		yes	1	451	500	93.80%	17	-	36533297	36577935	44639
		yes	1	441	500	92.80%	17	-	36511505	36528078	16574
		yes	1	455	500	95.00%	17	-	36549646	36550250	605
		yes	1	451	500	95.70%	17	-	36559002	36587927	28926
		yes	1	346	500	95.00%	17	-	36507427	36570454	63028
		yes	1	319	500	95.80%	17	-	36507589	36533885	26297
		yes	46	361	500	94.30%	17	-	36549867	36559453	9587
		yes	46	342	500	91.50%	17	-	36533808	36577846	44039
		yes	93	361	500	93.90%	17	-	36570128	36594420	24293
		yes	46	330	500	94.60%	17	-	36549996	36587850	37855
		yes	65	342	500	91.50%	17	+	36599972	36681331	81360
		yes	54	246	500	92.00%	17	-	36507452	36511922	4471
		yes	52	361	500	93.00%	17	-	36527710	36570040	42331
		yes	55	335	500	92.60%	17	+	36481880	36622889	141010
		yes	53	210	500	92.90%	17	+	36659881	36685925	26045
		yes	88	342	500	91.90%	17	+	36622526	36665391	42866
		yes	70	270	500	91.00%	17	+	36659520	36681406	21887
		yes	88	326	500	93.10%	17	+	36599848	36600365	518
		yes	69	342	500	90.30%	17	+	36636468	36659726	23259
		yes	204	311	500	92.60%	17	+	36482025	36665360	183336
		yes	123	303	500	91.80%	11	+	70927067	70927301	235
36,494,500	36,495,000	yes	1	500	500	92.60%	17	+	36515847	36516343	497
		yes	76	220	500	91.10%	17	-	36527239	36527383	145
		yes	101	220	500	93.40%	17	-	36507038	36507157	120
36,495,000	36,495,500	yes	6	500	500	92.50%	17	+	36516349	36516857	509
36,495,500	36,496,000	many interchromosomal									
36,496,000	36,496,500	many inter/intra									
36,496,500	36,497,000	many inter/intra									
36,497,000	36,497,500	yes	1	500	500	95.30%	17	+	36517027	36517534	508
		many inter/intra									
36,497,500	36,498,000	yes	1	500	500	89.50%	17	+	36517535	36518030	496
		many inter/intra									
36,498,000	36,498,500	yes	3	500	500	96.20%	17	+	36518001	36518502	502
		many inter/intra									
36,498,500	36,499,000	yes	1	500	500	95.00%	17	+	36518503	36519003	501
		many inter/intra									
36,499,000	36,499,500	yes	1	500	500	99.40%	17	+	36519004	36519503	500
		many inter/intra									
36,499,500	36,500,000	yes	1	500	500	98.60%	17	+	36519504	36520008	505
		many inter/intra									
36,500,000	36,500,500	yes	1	500	500	93.80%	17	+	36520009	36520516	508
		many inter/intra									
36,500,500	36,501,000	yes	1	486	500	93.20%	17	+	36520517	36521001	485
		many inter/intra									
36,501,000	36,501,500	yes	1	500	500	92.60%	17	+	36521310	36521804	495
		many inter/intra									
36,501,500	36,502,000	yes	1	500	500	91.40%	17	+	36521805	36522300	496
		many inter/intra									
36,502,000	36,502,500	yes	1	500	500	93.40%	17	+	36522301	36522794	494
		many inter/intra									
36,502,500	36,503,000	yes	1	500	500	92.10%	17	+	36522795	36523289	495
		many inter/intra									
36,503,000	36,503,500	yes	1	500	500	96.40%	17	+	36523290	36523794	505
		many inter/intra									
36,503,500	36,504,000	yes	1	500	500	94.20%	17	+	36523795	36524293	499
		many inter/intra									
36,504,000	36,504,500	yes	1	500	500	91.10%	17	+	36524294	36524791	498

36,504,500	36,505,000	many inter/intra yes	2	500	500	94.80%	17	+	36524793	36525292	500
36,505,000	36,505,500	many inter/intra yes	1	500	500	96.40%	17	+	36525293	36525788	496
36,505,500	36,506,000	many inter/intra yes	1	499	500	95.20%	17	+	36525789	36526287	499
36,506,000	36,506,500	many inter/intra yes	1	500	500	95.00%	17	+	36526289	36526787	499
36,506,500	36,507,000	many inter/intra yes	22	500	500	96.10%	17	+	36526721	36527201	481
36,507,000	36,507,500	yes	1	500	500	95.40%	17	+	36527202	36527700	499
		yes	21	500	500	91.90%	17	-	36515605	36516083	479
		yes	39	495	500	90.60%	17	-	36494098	36494719	622
		yes	308	500	500	96.40%	17	+	36533297	36533489	193
		yes	304	494	500	96.90%	17	+	36549646	36549956	311
		yes	308	500	500	95.90%	17	+	36559002	36559389	388
		yes	318	500	500	92.40%	17	+	36511505	36511807	303
36,507,500	36,508,000	yes	1	500	500	93.20%	17	+	36527701	36528230	530
		yes	1	497	500	93.60%	17	-	36493850	36515604	21755
		yes	1	500	500	92.70%	17	+	36527806	36594773	66968
		yes	10	497	500	91.40%	17	-	36515032	36515707	676
		yes	1	500	500	90.80%	17	+	36533610	36588083	54474
		yes	1	420	500	93.50%	17	+	36550095	36594680	44586
		yes	1	491	500	92.30%	17	+	36549858	36570696	20839
		yes	1	421	500	92.20%	17	+	36533490	36533958	469
		yes	29	372	500	90.80%	17	-	36481715	36665660	183946
		yes	10	377	500	93.20%	17	+	36511585	36596928	85344
		yes	10	307	500	93.40%	17	+	36533387	36570125	36739
		yes	1	303	500	91.70%	17	+	36570119	36577756	7638
		yes	33	307	500	93.10%	17	+	36511660	36533616	21957
		yes	93	297	500	94.70%	17	+	36511795	36594476	82682
		yes	18	396	500	90.30%	17	-	36665138	36681524	16387
		yes	1	288	500	94.50%	17	+	36559195	36594527	35333
		yes	2	285	500	91.50%	17	-	36481844	36515348	33505
		yes	16	307	500	91.00%	17	-	36481882	36636693	154812
		yes	1	266	500	92.00%	17	+	36587652	36596904	9253
		yes	52	279	500	94.00%	17	-	36659520	36681523	22004
		yes	17	154	500	92.40%	17	-	36481855	36600155	118301
		yes	48	168	500	95.10%	17	-	36515242	36681377	166136
		yes	1	164	500	97.80%	11	+	70971073	70971434	362
		yes	35	158	500	91.90%	17	-	36515417	36600266	84850
		yes	134	279	500	93.60%	17	-	36665185	36665660	476
		yes	169	279	500	92.80%	17	-	36681110	36681361	252
36,508,000	36,508,500	yes	1	500	500	95.00%	17	+	36528231	36528727	497
		yes	11	482	500	91.10%	17	-	36493368	36493839	472
		yes	11	482	500	90.90%	17	-	36514550	36515021	472
36,508,500	36,509,000	yes	1	500	500	93.70%	17	+	36528728	36529213	486
36,509,000	36,509,500	many inter/intra yes	1	500	500	99.00%	17	+	36529214	36529717	504
		yes	6	500	500	91.80%	17	-	36492660	36493152	493
		yes	6	500	500	92.80%	17	-	36513842	36514335	494
36,509,500	36,510,000	yes	1	500	500	96.00%	17	+	36529718	36530218	501
		yes	1	492	500	90.30%	17	-	36513349	36513841	493
		yes	1	492	500	90.30%	17	-	36513349	36513841	493
36,510,000	36,510,500	yes	1	500	500	95.20%	17	+	36530219	36530718	500
36,510,500	36,511,000	yes	1	500	500	95.50%	17	+	36530719	36531230	512
36,511,000	36,511,500	many inter/intra yes	1	358	500	94.80%	17	+	36531231	36531578	348
36,511,500	36,512,000	many inter/intra yes	6	467	500	94.20%	17	+	36533307	36550130	16824
		yes	1	467	500	93.80%	17	+	36527317	36570259	42943
		yes	6	467	500	92.60%	17	+	36507317	36570154	62838
		yes	6	459	500	92.10%	17	+	36559012	36587798	28787
		yes	1	441	500	91.70%	17	+	36549622	36594473	44852
		yes	6	467	500	90.40%	17	-	36494132	36515787	21656
		yes	101	423	500	91.40%	17	+	36559302	36577882	18581
		yes	146	467	500	92.40%	17	+	36507460	36559230	51771
		yes	144	401	500	90.40%	17	-	36481811	36494269	12459
		yes	143	422	500	92.80%	17	+	36569806	36594458	24653
		yes	162	441	500	91.30%	17	-	36599917	36665655	65739
		yes	283	467	500	91.60%	17	+	36527780	36549998	22219
		yes	267	463	500	92.60%	17	+	36527899	36577528	49630
		yes	161	399	500	90.30%	17	+	36570151	36577843	7693
		yes	162	423	500	90.30%	17	-	36515280	36636939	121660
		yes	177	320	500	92.20%	17	-	36494291	36600096	105806
		yes	297	467	500	95.50%	17	+	36527689	36527946	258
		yes	222	414	500	94.70%	17	-	36494107	36636939	142833

		yes	282	441	500	94.90%	17	-	36665344	36681376	16033
		yes	172	307	500	97.00%	17	-	36515276	36515516	241
36,512,000	36,512,500	yes	1	500	500	94.90%	17	+	36490610	36491328	719
		many inter/intra									
36,512,500	36,513,000	yes	1	500	500	99.20%	17	+	36491329	36491827	499
		many inter/intra									
36,513,000	36,513,500	yes	1	500	500	92.50%	17	+	36491828	36492318	491
		many inter/intra									
36,513,500	36,514,000	yes	1	500	500	93.00%	17	+	36492319	36492817	499
		yes	1	500	500	92.20%	17	-	36509342	36509840	499
		yes	1	500	500	91.60%	17	-	36529560	36530060	501
36,514,000	36,514,500	yes	1	500	500	98.80%	17	+	36492818	36493317	500
		yes	1	500	500	91.90%	17	-	36508532	36509345	814
		yes	1	500	500	92.30%	17	-	36528760	36529559	800
36,514,500	36,515,000	yes	1	500	500	98.80%	17	+	36493318	36493817	500
		yes	328	483	500	94.90%	17	-	36528278	36528434	157
36,515,000	36,515,500	yes	1	496	500	96.70%	17	+	36493818	36494328	511
		yes	1	494	500	91.20%	17	-	36507476	36528262	20787
		yes	65	500	500	91.30%	17	-	36533580	36588044	54465
		yes	105	496	500	91.10%	17	-	36533464	36559608	26145
		yes	140	496	500	90.50%	17	-	36559169	36587970	28802
		yes	159	493	500	91.70%	17	-	36570021	36594641	24621
		yes	234	493	500	92.80%	17	+	36494306	36642548	148243
		yes	159	497	500	94.80%	17	+	36481715	36494254	12540
		yes	228	496	500	90.40%	17	-	36549832	36577756	27925
		yes	135	498	500	91.50%	17	+	36636407	36675444	39038
		yes	135	405	500	92.70%	17	+	36659473	36681566	22094
		yes	275	455	500	92.60%	17	-	36570134	36594540	24407
		yes	228	500	500	94.30%	17	-	36436733	36570377	133645
		yes	236	467	500	92.80%	17	+	36515610	36659726	144117
		yes	235	498	500	91.30%	17	+	36648212	36681377	33166
		yes	237	481	500	91.00%	17	+	36481880	36600428	118549
		yes	235	467	500	94.50%	17	+	36642662	36665670	23009
		yes	234	355	500	95.00%	17	-	36511927	36570040	58114
		yes	264	458	500	95.80%	17	+	36636466	36642498	6033
		yes	247	498	500	88.60%	11	+	1675128	1675412	285
36,515,500	36,516,000	yes	4	500	500	94.20%	17	-	36527306	36559386	32081
		yes	4	500	500	93.60%	17	-	36507105	36507601	497
		yes	5	500	500	93.30%	17	+	36481995	36494652	12658
		yes	4	298	500	95.60%	17	-	36533297	36549854	16558
		yes	1	302	500	94.80%	17	-	36549646	36577572	27927
		yes	6	298	500	93.90%	17	-	36559002	36577867	18866
		yes	6	288	500	90.50%	17	-	36511505	36587871	76367
		yes	34	301	500	90.80%	17	-	36569743	36577884	8142
		yes	36	177	500	91.30%	17	+	36494173	36681553	187381
		yes	111	304	500	94.70%	17	-	36587527	36594564	7038
		yes	43	191	500	93.80%	17	-	36570018	36577740	7723
		yes	36	178	500	91.50%	17	-	36569896	36577567	7672
		yes	56	192	500	94.80%	17	-	36577636	36594397	16762
		yes	31	182	500	94.50%	17	+	36665425	36681412	15988
		yes	51	191	500	92.70%	17	+	36665271	36665672	402
		many inter/intra									
36,516,000	36,516,500	yes	1	496	500	91.90%	17	+	36494653	36495137	485
36,516,500	36,517,000	yes	1	500	500	93.00%	17	+	36495140	36496972	1833
36,517,000	36,517,500	yes	1	500	500	94.40%	17	+	36496973	36497464	492
		many inter/intra									
36,517,500	36,518,000	yes	1	500	500	90.80%	17	+	36497465	36498001	537
		many inter/intra									
36,518,000	36,518,500	yes	2	500	500	95.00%	17	+	36498002	36498496	495
		many inter/intra									
36,518,500	36,519,000	yes	1	500	500	94.40%	17	+	36498497	36498995	499
		many inter/intra									
36,519,000	36,519,500	yes	1	500	500	99.40%	17	+	36498996	36499495	500
		many inter/intra									
36,519,500	36,520,000	yes	1	500	500	97.60%	17	+	36499496	36499990	495
		many inter/intra									
36,520,000	36,520,500	yes	1	500	500	92.20%	17	+	36499991	36500482	492
		many inter/intra									
36,520,500	36,521,000	yes	4	500	500	93.80%	17	+	36500486	36500983	498
		many inter/intra									
36,521,000	36,521,500	many inter/intra									
36,521,500	36,522,000	yes	1	500	500	93.80%	17	+	36501196	36501698	503
		many inter/intra									
36,522,000	36,522,500	yes	2	500	500	92.80%	17	+	36501700	36502198	499
		many inter/intra									
36,522,500	36,523,000	yes	1	500	500	94.40%	17	+	36502199	36502707	509
		many inter/intra									

36,523,000	36,523,500	yes	1	500	500	93.60%	17	+	36502708	36503204	497
36,523,500	36,524,000	many inter/intra yes	1	500	500	95.80%	17	+	36503205	36503706	502
36,524,000	36,524,500	many inter/intra yes	1	500	500	93.40%	17	+	36503707	36504205	499
36,524,500	36,525,000	many inter/intra yes	18	500	500	93.60%	17	+	36504223	36504706	484
36,525,000	36,525,500	many inter/intra yes	1	500	500	95.60%	17	+	36504707	36505209	503
36,525,500	36,526,000	many inter/intra yes	7	500	500	96.00%	17	+	36505216	36505710	495
36,526,000	36,526,500	many inter/intra yes	1	500	500	95.80%	17	+	36505711	36506210	500
36,526,500	36,527,000	many inter/intra yes	1	500	500	97.40%	17	+	36506211	36506799	589
36,527,000	36,527,500	yes	1	500	500	94.40%	17	+	36506800	36507298	499
36,527,500	36,528,000	yes	1	500	500	95.80%	17	+	36507299	36587860	80562
		yes	1	500	500	95.00%	17	-	36494079	36515805	21727
		yes	9	500	500	95.40%	17	+	36533297	36550051	16755
		yes	5	500	500	95.40%	17	+	36549646	36559283	9638
		yes	9	500	500	93.10%	17	+	36559002	36570312	11311
		yes	19	500	500	93.30%	17	+	36511505	36533818	22314
		yes	1	495	500	94.40%	17	-	36481805	36494458	12654
		yes	7	500	500	90.90%	17	-	36515261	36681613	166353
		yes	99	500	500	95.20%	17	+	36533499	36570387	36889
		yes	114	500	500	90.50%	17	+	36559302	36587770	28469
		yes	114	500	500	92.10%	17	+	36533822	36587620	53799
		yes	116	500	500	90.70%	17	+	36570018	36577676	7659
		yes	119	474	500	90.90%	17	-	36515242	36681537	166296
		yes	281	500	500	95.60%	17	+	36511782	36550183	38402
		yes	190	500	500	93.00%	17	+	36570047	36577766	7720
		yes	159	390	500	90.30%	17	-	36482011	36681512	199502
		yes	118	451	500	92.40%	17	-	36599844	36665391	65548
		yes	129	467	500	93.70%	17	-	36482039	36600142	118104
		yes	341	500	500	93.90%	17	-	36659545	36659944	400
		yes	370	500	500	93.00%	17	-	36681135	36681376	242
36,528,000	36,528,500	many inter/intra yes	4	500	500	94.60%	17	+	36507547	36508269	723
		yes	1	500	500	90.40%	17	-	36514764	36515260	497
		yes	1	500	500	90.40%	17	-	36493582	36494078	497
36,528,500	36,529,000	yes	1	500	500	95.40%	17	+	36508270	36508785	516
36,529,000	36,529,500	many inter/intra yes	1	500	500	97.20%	17	+	36508786	36509285	500
		yes	114	431	500	93.40%	17	-	36492940	36493258	319
		yes	114	431	500	92.60%	17	-	36514121	36514440	320
36,529,500	36,530,000	yes	1	500	500	97.20%	17	+	36509286	36509780	495
		yes	1	500	500	91.70%	17	-	36513560	36514059	500
		yes	1	500	500	90.40%	17	-	36492379	36492877	499
36,530,000	36,530,500	yes	1	499	500	95.20%	17	+	36509781	36510279	499
36,530,500	36,531,000	yes	1	500	500	93.60%	17	+	36510281	36510776	496
36,531,000	36,531,500	many inter/intra yes	1	500	500	95.30%	17	+	36510777	36511269	493
36,531,500	36,532,000	many inter/intra									
36,532,000	36,532,500	many inter/intra									
36,532,500	36,533,000	nothing									
36,533,000	36,533,500	yes	296	500	500	97.60%	17	+	36559000	36587661	28662
		yes	298	500	500	97.60%	17	+	36527508	36527920	413
		yes	298	500	500	97.60%	17	-	36494143	36515797	21655
		yes	298	500	500	97.60%	17	+	36549650	36549972	323
		yes	298	500	500	96.60%	17	+	36507307	36507509	203
		yes	308	500	500	95.90%	17	+	36511505	36511712	208
		yes	298	487	500	98.90%	17	-	36494051	36494450	400
36,533,500	36,534,000	yes	1	500	500	96.70%	17	+	36549973	36588040	38068
		yes	1	500	500	94.20%	17	+	36507690	36570567	62878
		yes	1	439	500	96.40%	17	+	36511586	36587980	76395
		yes	1	500	500	93.10%	17	-	36493887	36515594	21708
		yes	1	500	500	93.60%	17	+	36507615	36528190	20576
		yes	24	500	500	91.80%	17	-	36515069	36622883	107815
		yes	1	434	500	95.00%	17	+	36527921	36559578	31658
		yes	25	408	500	91.70%	17	-	36481717	36659944	178228
		yes	1	355	500	95.70%	17	+	36559205	36577892	18688
		yes	1	345	500	96.20%	17	+	36527711	36577747	50037
		yes	24	339	500	93.20%	17	-	36515361	36665655	150295
		yes	1	345	500	94.70%	17	+	36549868	36577837	27970
		yes	24	345	500	95.30%	17	+	36527659	36570368	42710

		yes	6	275	500	94.70%	17	+	36570314	36577782	7469
		yes	162	345	500	97.60%	17	+	36507476	36511802	4327
		yes	7	203	500	94.10%	17	-	36482077	36600155	118079
		yes	1	217	500	94.10%	17	-	36481899	36494292	12394
36,534,000	36,534,500	yes	112	332	500	95.20%	10	+	72321401	72321829	429
36,534,500	36,535,000	yes	274	490	500	94.80%	5	-	89767996	89768214	219
36,535,000	36,535,500	many inter/intra									
36,535,500	36,536,000	many inter/intra									
36,536,000	36,536,500	nothing									
36,536,500	36,537,000	many inter/intra									
36,537,000	36,537,500	many inter/intra									
36,537,500	36,538,000	many inter/intra									
36,538,000	36,538,500	many inter/intra									
36,538,500	36,539,000	many inter/intra									
36,539,000	36,539,500	many inter/intra									
36,539,500	36,540,000	nothing									
36,540,000	36,540,500	nothing									
36,540,500	36,541,000	many inter/intra									
36,541,000	36,541,500	many inter/intra									
36,541,500	36,542,000	nothing									
36,542,000	36,542,500	many interchromosomal									
36,542,500	36,543,000	yes	133	343	500	93.20%	12	-	25342119	25342625	507
36,543,000	36,543,500	many inter/intra									
36,543,500	36,544,000	many inter/intra									
36,544,000	36,544,500	nothing									
36,544,500	36,545,000	many inter/intra									
36,545,000	36,545,500	many inter/intra									
36,545,500	36,546,000	many inter/intra									
36,546,000	36,546,500	many inter/intra									
36,546,500	36,547,000	many inter/intra									
36,547,000	36,547,500	nothing									
36,547,500	36,548,000	many inter/intra									
36,548,000	36,548,500	nothing									
36,548,500	36,549,000	nothing									
36,549,000	36,549,500	nothing									
36,549,500	36,550,000	yes	147	500	500	96.10%	17	+	36507303	36559231	51929
		yes	147	493	500	95.10%	17	+	36527504	36559419	31916
		yes	141	500	500	93.00%	17	+	36558990	36587688	28699
		yes	147	500	500	94.40%	17	-	36515433	36515801	369
		yes	151	500	500	96.00%	17	+	36533297	36533646	350
		yes	147	500	500	90.80%	17	-	36494131	36494454	324
		yes	161	500	500	91.80%	17	+	36511505	36511844	340
		yes	243	500	500	91.40%	17	+	36436431	36507716	71286
		yes	280	500	500	92.60%	17	+	36507451	36527947	20497
		yes	300	500	500	92.90%	17	+	36436842	36570215	133374
		yes	287	472	500	91.20%	17	-	36599860	36665613	65754
36,550,000	36,550,500	yes	1	410	500	90.00%	17	+	36533647	36594776	61130
36,550,500	36,551,000	yes	1	410	500	90.00%	17	+	36533647	36594776	61130
		yes	1	410	500	92.50%	17	+	36511725	36578080	66356
		yes	1	358	500	90.80%	17	+	36533527	36588034	54508
		yes	1	336	500	93.70%	17	+	36507432	36578020	70589
		yes	1	310	500	94.10%	17	-	36493941	36515312	21372
		yes	1	275	500	91.30%	17	-	36481715	36675574	193860
		yes	1	298	500	94.10%	17	+	36527738	36596948	69211
		yes	13	356	500	91.30%	17	-	36636349	36665375	29027
		yes	1	215	500	93.70%	17	+	36559232	36594569	35338
		yes	13	209	500	93.60%	17	-	36481880	36599891	118012
		yes	29	356	500	91.90%	17	-	36665080	36681566	16487
		yes	28	210	500	93.30%	17	-	36515610	36659569	143960
		yes	1	215	500	93.00%	17	+	36570261	36596851	26591
		yes	54	356	500	92.20%	17	-	36659415	36681598	22184
		yes	34	232	500	91.70%	17	-	36648182	36665621	17440
		yes	83	212	500	98.50%	17	+	36507592	36570370	62779
		yes	1	207	500	91.20%	17	+	36577445	36594300	16856
		yes	1	210	500	91.30%	17	+	36587569	36594387	6819
		yes	34	194	500	93.70%	17	-	36636454	36642707	6254
		yes	1	212	500	92.60%	17	+	36570006	36577749	7744
		yes	27	299	500	92.40%	17	-	36681063	36681553	491
		yes	92	212	500	96.50%	17	+	36507496	36577659	70164
		yes	1	167	500	97.00%	17	+	36527633	36594566	66934
		yes	1	114	500	90.50%	17	-	36482065	36494130	12066
		yes	29	199	500	93.10%	17	-	36599972	36648262	48291
		yes	57	228	500	95.00%	11	-	1675096	1675411	316
36,551,000	36,551,500	nothing									
36,551,500	36,552,000	many inter/intra									
36,552,000	36,552,500	many inter/intra									

36,552,500	36,553,000	many inter/intra									
36,553,000	36,553,500	many inter/intra									
36,553,500	36,554,000	nothing									
36,554,000	36,554,500	many inter/intra									
36,554,500	36,555,000	many inter/intra									
36,555,000	36,555,500	nothing									
36,555,500	36,556,000	yes	60	165	500	91.90%	18	+	58830215	58830323	109
36,556,000	36,556,500	nothing									
36,556,500	36,557,000	nothing									
36,557,000	36,557,500	nothing									
36,557,500	36,558,000	many inter/intra									
36,558,000	36,558,500	many inter/intra									
36,558,500	36,559,000	nothing									
36,559,000	36,559,500	yes	1	500	500	96.60%	17	+	36533295	36550219	16925
		yes	1	500	500	96.00%	17	+	36507305	36533854	26550
		yes	1	496	500	95.90%	17	-	36494047	36515799	21753
		yes	1	500	500	93.60%	17	+	36549648	36577892	28245
		yes	1	490	500	95.60%	17	+	36527506	36570368	42863
		yes	13	500	500	92.90%	17	+	36511505	36587896	76392
		yes	1	490	500	90.90%	17	-	36481795	36494452	12658
		yes	93	481	500	92.20%	17	+	36533499	36587757	54259
		yes	110	500	500	91.70%	17	+	36507549	36528035	20487
		yes	148	490	500	93.40%	17	+	36533516	36570173	36658
		yes	112	490	500	92.60%	17	+	36569792	36587646	17855
		yes	112	484	500	93.30%	17	-	36515361	36636675	121315
		yes	304	500	500	94.50%	17	+	36507413	36507804	392
		yes	114	317	500	96.70%	17	+	36569764	36587758	17995
		yes	274	490	500	93.10%	17	+	36507593	36533724	26132
		yes	229	454	500	91.30%	17	-	36515227	36600141	84915
		yes	95	267	500	90.80%	17	-	36481787	36482072	286
		yes	260	490	500	92.50%	17	-	36636861	36659569	22709
		yes	154	359	500	92.50%	17	-	36481902	36494269	12368
		yes	281	479	500	96.90%	17	-	36622695	36636905	14211
36,559,500	36,560,000	yes	12	188	500	94.70%	17	+	36587908	36594783	6876
		yes	12	181	500	93.10%	17	+	36533866	36550408	16543
36,560,000	36,560,500	nothing									
36,560,500	36,561,000	many inter/intra									
36,561,000	36,561,500	many inter/intra									
36,561,500	36,562,000	many inter/intra									
36,562,000	36,562,500	many inter/intra									
36,562,500	36,563,000	many inter/intra									
36,563,000	36,563,500	many inter/intra									
36,563,500	36,564,000	many inter/intra									
36,564,000	36,564,500	many inter/intra									
36,564,500	36,565,000	nothing									
36,565,000	36,565,500	many inter/intra									
36,565,500	36,566,000	many inter/intra									
36,566,000	36,566,500	many inter/intra									
36,566,500	36,567,000	nothing									
36,567,000	36,567,500	nothing									
36,567,500	36,568,000	yes	371	500	500	93.90%	20	+	3704783	3705001	219
36,568,000	36,568,500	many inter/intra									
36,568,500	36,569,000	many inter/intra									
36,569,000	36,569,500	nothing									
36,569,500	36,570,000	yes	245	454	500	90.30%	17	-	36600328	36659963	59636
		yes	265	478	500	91.70%	17	-	36647995	36681536	33542
		yes	286	454	500	91.00%	17	+	36451019	36559284	108266
		yes	286	454	500	91.00%	17	+	36444365	36570313	125949
		yes	265	463	500	90.10%	17	-	36636598	36642682	6085
		yes	265	454	500	93.60%	17	-	36636478	36647983	11506
36,570,000	36,570,500	yes	1	500	500	96.20%	17	+	36527942	36587972	60031
		yes	1	500	500	93.50%	17	+	36533521	36559575	26055
		yes	5	500	500	92.00%	17	+	36507535	36533930	26396
		yes	18	500	500	94.30%	17	+	36511781	36550295	38515
		yes	1	492	500	93.60%	17	+	36549889	36594654	44766
		yes	19	500	500	90.60%	17	+	36511662	36577980	66319
		yes	5	500	500	91.30%	17	-	36493955	36515569	21615
		yes	18	499	500	90.50%	17	-	36515138	36681595	166458
		yes	19	484	500	92.90%	17	+	36507414	36528107	20694
		yes	60	479	500	91.00%	17	-	36481715	36515589	33875
		yes	19	396	500	91.50%	17	+	36549757	36570065	20309
		yes	1	371	500	93.50%	17	+	36570330	36594566	24237
		yes	21	484	500	90.00%	17	+	36507476	36507876	401
		yes	79	408	500	90.70%	17	-	36515361	36659944	144584
		yes	77	404	500	93.90%	17	+	36511660	36577587	65928
		yes	19	341	500	91.70%	17	+	36527615	36577884	50270

		yes	98	414	500	93.90%	17	+	36511906	36549852	37947
		yes	78	383	500	94.10%	17	-	36599701	36665655	65955
		yes	1	274	500	92.60%	17	+	36559226	36594283	35058
		yes	221	488	500	93.20%	17	-	36659482	36681413	21932
		yes	274	414	500	90.10%	17	+	36511662	36511802	141
		yes	262	500	500	92.90%	17	-	36665141	36665450	310
		yes	240	378	500	96.00%	17	-	36515227	36665409	150183
		yes	17	369	500	96.40%	17	-	36494233	36515257	21025
		yes	112	390	500	94.60%	17	-	36515274	36599933	84660
36,570,500	36,571,000	yes	1	412	500	91.30%	17	+	36587973	36588385	413
36,571,000	36,571,500	many inter/intra									
36,571,500	36,572,000	many inter/intra									
36,572,000	36,572,500	nothing									
36,572,500	36,573,000	nothing									
36,573,000	36,573,500	nothing									
36,573,500	36,574,000	nothing									
36,574,000	36,574,500	nothing									
36,574,500	36,575,000	yes	1	499	499	96.80%	9	-	30794205	30794697	493
36,575,000	36,575,500	yes	1	500	500	98.00%	9	-	30793699	30794203	505
36,575,500	36,576,000	many inter/intra									
36,576,000	36,576,500	many inter/intra									
36,576,500	36,577,000	nothing									
36,577,000	36,577,500	yes	364	469	500	92.10%	17	+	36587532	36587712	181
36,577,500	36,578,000	yes	1	479	500	94.50%	17	+	36533477	36587970	54494
		yes	48	465	500	90.60%	17	+	36507414	36507876	463
		yes	34	383	500	92.10%	17	+	36527661	36587646	59986
		yes	34	383	500	90.00%	17	+	36527631	36533604	5974
		yes	34	383	500	91.90%	17	+	36533525	36570368	36844
		yes	1	385	500	94.00%	17	-	36515668	36681527	165860
		yes	1	263	500	93.60%	17	+	36559182	36587856	28675
		yes	152	441	500	90.60%	17	-	36636698	36681145	44448
		yes	227	353	500	96.30%	17	-	36515430	36599892	84463
		yes	331	482	500	92.40%	17	-	36681065	36681290	226
		yes	62	193	500	93.20%	17	-	36515425	36515688	264
36,578,000	36,578,500	nothing									
36,578,500	36,579,000	many inter/intra									
36,579,000	36,579,500	many inter/intra									
36,579,500	36,580,000	nothing									
36,580,000	36,580,500	nothing									
36,580,500	36,581,000	nothing									
36,581,000	36,581,500	nothing									
36,581,500	36,582,000	nothing									
36,582,000	36,582,500	many inter/intra									
36,582,500	36,583,000	many inter/intra									
36,583,000	36,583,500	many inter/intra									
36,583,500	36,584,000	nothing									
36,584,000	36,584,500	nothing									
36,584,500	36,585,000	nothing									
36,585,000	36,585,500	nothing									
36,585,500	36,586,000	nothing									
36,586,000	36,586,500	nothing									
36,586,500	36,587,000	nothing									
36,587,000	36,587,500	nothing									
36,587,500	36,588,000	yes	28	478	500	93.00%	17	+	36507301	36550300	43000
		yes	33	471	500	95.00%	17	+	36549648	36577978	28331
		yes	28	496	500	91.80%	17	-	36493923	36515803	21881
		yes	66	476	500	93.80%	17	+	36511721	36559578	47858
		yes	32	496	500	91.00%	17	-	36515105	36665674	150570
		yes	65	465	500	93.20%	17	+	36507592	36594654	87063
		yes	130	457	500	92.60%	17	+	36507477	36507876	400
		yes	33	387	500	92.80%	17	+	36569744	36594330	24587
		yes	32	422	500	93.20%	17	-	36515335	36685986	170652
		yes	53	387	500	92.30%	17	+	36527619	36577747	50129
		yes	37	342	500	91.40%	17	+	36549802	36577747	27946
		yes	66	377	500	93.60%	17	+	36507533	36577587	70055
		yes	53	343	500	91.00%	17	+	36549761	36577508	27748
		yes	98	386	500	92.50%	17	-	36600304	36685938	85635
		yes	61	335	500	95.20%	17	+	36570162	36594296	24135
		yes	66	317	500	93.30%	17	+	36527839	36577782	49944
		yes	188	387	500	95.00%	17	-	36636627	36681404	44778
		yes	188	491	500	94.00%	17	-	36665139	36681230	16092
		yes	145	461	500	93.40%	17	-	36685459	36685951	493
		yes	65	236	500	95.40%	17	+	36570031	36577761	7731
		yes	128	389	500	92.40%	17	-	36515608	36665599	149992
		yes	122	328	500	93.60%	17	-	36482039	36636754	154716
36,588,000	36,588,500	nothing									

36,588,500	36,589,000	nothing									
36,589,000	36,589,500	nothing									
36,589,500	36,590,000	nothing									
36,590,000	36,590,500	many inter/intra									
36,590,500	36,591,000	nothing									
36,591,000	36,591,500	many inter/intra									
36,591,500	36,592,000	many inter/intra									
36,592,000	36,592,500	many inter/intra									
36,592,500	36,593,000	nothing									
36,593,000	36,593,500	nothing									
36,593,500	36,594,000	nothing									
36,594,000	36,594,500	yes	209	459	500	91.00%	17	+	36559469	36577836	18368
		yes	206	474	500	90.50%	17	-	36515461	36681378	165918
		yes	268	477	500	91.00%	17	+	36527734	36570370	42637
		yes	323	459	500	95.50%	17	+	36507561	36549926	42366
		yes	267	479	500	91.10%	17	-	36600071	36681233	81163
		yes	240	482	500	93.00%	17	-	36515498	36600337	84840
		yes	207	358	500	92.10%	17	+	36559242	36587643	28402
36,594,500	36,595,000	yes	1	281	500	93.20%	17	+	36533525	36597066	63542
		yes	1	284	500	91.70%	17	+	36533420	36559687	26268
		yes	45	276	500	91.10%	17	+	36507654	36508001	348
36,595,000	36,595,500	nothing									
36,595,500	36,596,000	nothing									
36,596,000	36,596,500	nothing									
36,596,500	36,597,000	yes	242	449	500	92.20%	17	+	36559294	36570501	11208
		yes	248	449	500	91.70%	17	+	36569894	36587990	18097
		yes	319	423	500	91.70%	17	+	36451019	36451183	165
36,597,000	36,597,500	nothing									
36,597,500	36,598,000	many inter/intra									
36,598,000	36,598,500	many inter/intra									
36,598,500	36,599,000	many inter/intra									
36,599,000	36,599,500	many inter/intra									
36,599,500	36,600,000	yes	38	488	500	94.80%	17	+	36622229	36681411	59183
		yes	74	494	500	96.20%	17	+	36636343	36659956	23614
		yes	77	488	500	96.60%	17	+	36659413	36685783	26371
		yes	78	489	500	94.60%	17	+	36675071	36681601	6531
		yes	77	479	500	95.00%	17	+	36647744	36665462	17719
		yes	77	489	500	93.80%	17	+	36642194	36648297	6104
		yes	78	449	500	94.60%	17	+	36622399	36648098	25700
		yes	77	433	500	94.20%	17	+	36665078	36690562	25485
		yes	175	449	500	93.70%	17	+	36600004	36659767	59764
		yes	74	457	500	93.00%	17	+	36685386	36690484	5099
		yes	202	482	500	92.80%	17	-	36569838	36577761	7924
		yes	217	488	500	90.10%	17	+	36622523	36648062	25540
		yes	202	482	500	90.60%	17	-	36559229	36570067	10839
		yes	202	486	500	93.40%	17	-	36533580	36559308	25729
		yes	117	396	500	90.10%	17	-	36436673	36507900	71228
		yes	299	437	500	92.60%	17	-	36577502	36718445	140944
		yes	210	394	500	93.40%	17	-	36507562	36511898	4337
36,600,000	36,600,500	yes	1	415	500	90.40%	17	+	36636567	36681331	44765
		yes	1	415	500	90.70%	17	+	36647949	36681523	33575
		yes	46	415	500	91.50%	17	+	36636483	36659726	23244
		yes	46	431	500	90.10%	17	-	36577485	36587856	10372
		yes	61	436	500	91.90%	17	-	36570071	36594504	24434
		yes	76	397	500	92.50%	17	-	36549819	36577867	28049
		yes	57	401	500	91.00%	17	-	36511642	36559388	47747
		yes	222	414	500	92.80%	17	+	36599762	36642506	42745
		yes	4	149	500	93.20%	17	+	36685496	36690589	5094
		yes	55	156	500	94.90%	17	-	36436827	36511898	75072
		yes	319	442	500	91.50%	17	-	36587721	36594549	6829
		yes	81	332	500	92.20%	17	-	36451019	36570123	119105
		yes	55	268	500	94.00%	17	-	36511736	36528001	16266
		yes	132	402	500	92.20%	17	-	36577574	36577871	298
36,600,500	36,601,000	nothing									
36,601,000	36,601,500	many inter/intra									
36,601,500	36,602,000	many inter/intra									
36,602,000	36,602,500	nothing									
36,602,500	36,603,000	many inter/intra									
36,603,000	36,603,500	nothing									
36,603,500	36,604,000	many inter/intra									
36,604,000	36,604,500	many inter/intra									
36,604,500	36,605,000	many inter/intra									
36,605,000	36,605,500	many inter/intra									
36,605,500	36,606,000	nothing									
36,606,000	36,606,500	many inter/intra									
36,606,500	36,607,000	nothing									

36,607,000	36,607,500	nothing									
36,607,500	36,608,000	nothing									
36,608,000	36,608,500	many inter/intra									
36,608,500	36,609,000	many inter/intra									
36,609,000	36,609,500	many inter/intra									
36,609,500	36,610,000	many inter/intra									
36,610,000	36,610,500	many inter/intra									
36,610,500	36,611,000	many inter/intra									
36,611,000	36,611,500	many inter/intra									
36,611,500	36,612,000	many inter/intra									
36,612,000	36,612,500	many inter/intra									
36,612,500	36,613,000	many inter/intra									
36,613,000	36,613,500	many inter/intra									
36,613,500	36,614,000	many inter/intra									
36,614,000	36,614,500	many inter/intra									
36,614,500	36,615,000	many inter/intra									
36,615,000	36,615,500	many inter/intra									
36,615,500	36,616,000	many inter/intra									
36,616,000	36,616,500	many inter/intra									
36,616,500	36,617,000	many inter/intra									
36,617,000	36,617,500	many inter/intra									
36,617,500	36,618,000	many inter/intra									
36,618,000	36,618,500	many inter/intra									
36,618,500	36,619,000	many inter/intra									
36,619,000	36,619,500	many inter/intra									
36,619,500	36,620,000	many inter/intra									
36,620,000	36,620,500	nothing									
36,620,500	36,621,000	nothing									
36,621,000	36,621,500	nothing									
36,621,500	36,622,000	nothing									
36,622,000	36,622,500	yes	384	495	500	90.90%	17	+	36647728	36647840	113
		yes	400	500	500	91.60%	17	+	36599577	36599680	104
36,622,500	36,623,000	yes	1	500	500	90.40%	17	+	36659515	36681522	22008
		yes	24	389	500	90.60%	17	+	36636484	36665660	29177
		yes	38	500	500	92.30%	17	+	36515550	36648308	132759
		yes	1	390	500	92.90%	17	+	36647846	36675584	27739
		yes	6	389	500	92.90%	17	+	36600076	36659647	59572
		yes	6	394	500	90.60%	17	-	36450923	36559473	108551
		yes	189	389	500	93.20%	17	+	36599877	36647963	48087
		yes	196	389	500	90.20%	17	+	36515322	36600404	85083
		yes	24	294	500	92.30%	17	-	36511721	36533573	21853
		yes	195	389	500	93.50%	17	+	36647947	36659716	11770
		yes	6	219	500	95.20%	17	-	36436675	36511940	75266
		yes	38	217	500	95.80%	11	-	70971092	70971394	303
36,623,000	36,623,500	many inter/intra									
36,623,500	36,624,000	many inter/intra									
36,624,000	36,624,500	nothing									
36,624,500	36,625,000	many inter/intra									
36,625,000	36,625,500	many inter/intra									
36,625,500	36,626,000	many inter/intra									
36,626,000	36,626,500	many inter/intra									
36,626,500	36,627,000	many inter/intra									
36,627,000	36,627,500	many inter/intra									
36,627,500	36,628,000	many inter/intra									
36,628,000	36,628,500	many inter/intra									
36,628,500	36,629,000	many inter/intra									
36,629,000	36,629,500	many inter/intra									
36,629,500	36,630,000	many inter/intra									
36,630,000	36,630,500	many inter/intra									
36,630,500	36,631,000	many inter/intra									
36,631,000	36,631,500	many inter/intra									
36,631,500	36,632,000	many inter/intra									
36,632,000	36,632,500	many inter/intra									
36,632,500	36,633,000	many inter/intra									
36,633,000	36,633,500	many inter/intra									
36,633,500	36,634,000	nothing									
36,634,000	36,634,500	nothing									
36,634,500	36,635,000	nothing									
36,635,000	36,635,500	many inter/intra									
36,635,500	36,636,000	many inter/intra									
36,636,000	36,636,500	yes	6	500	500	93.70%	17	+	36664734	36665344	611
		yes	6	500	500	92.20%	17	+	36659080	36681182	22103
		yes	6	500	500	90.10%	17	+	36647412	36675351	27940
		yes	56	500	500	94.50%	17	+	36641901	36659565	17665
		yes	22	500	500	91.60%	17	+	36674740	36685656	10917
		yes	284	500	500	94.40%	17	+	36647680	36647908	229
		yes	344	500	500	94.80%	17	+	36599573	36599758	186

		yes	350	500	500	92.50%	17	-	36533678	36550355	16678
		yes	295	500	500	96.00%	17	-	36559263	36559681	419
36,636,500	36,637,000	yes	1	500	500	98.20%	17	+	36599732	36648351	48620
		yes	1	494	500	96.80%	17	+	36665216	36665709	494
		yes	1	500	500	96.30%	17	+	36642332	36660005	17674
		yes	1	498	500	94.20%	17	+	36681156	36681690	535
		yes	1	467	500	94.90%	17	+	36685528	36685993	466
		yes	1	455	500	95.80%	17	+	36675223	36685703	10481
		yes	1	494	500	95.10%	17	+	36647882	36675674	27793
		yes	20	445	500	90.70%	17	+	36515259	36659716	144458
		yes	1	445	500	96.30%	17	+	36659566	36681411	21846
		yes	25	208	500	94.80%	17	+	36636668	36648074	11407
		yes	56	456	500	91.40%	17	+	36515550	36600415	84866
		yes	22	445	500	92.90%	17	-	36444520	36570117	125598
		yes	25	214	500	94.90%	17	+	36622801	36647951	25151
36,637,000	36,637,500	yes	1	500	500	100.00%	17	+	36637000	36637499	500
		yes	1	500	500	92.20%	17	+	36642802	36643301	500
		yes	1	500	500	91.40%	17	+	36660006	36660505	500
		yes	1	495	500	90.10%	17	+	36665716	36682146	16431
		yes	298	496	500	90.50%	17	+	36666011	36666210	200
36,637,500	36,638,000	yes	1	500	500	95.50%	17	+	36648852	36649351	500
36,638,000	36,638,500	nothing									
36,638,500	36,639,000	yes	2	495	500	91.10%	17	+	36661502	36661998	497
		yes	1	493	500	90.70%	17	+	36655972	36656465	494
		yes	1	493	500	90.10%	17	+	36644292	36644786	495
		yes	17	500	500	90.30%	18	-	28462318	28462803	486
36,639,000	36,639,500	many inter/intra									
36,639,500	36,640,000	yes	6	481	500	92.00%	17	+	36662207	36662683	477
		yes	11	480	500	91.90%	17	+	36644994	36657146	12153
		yes	173	480	500	90.60%	17	+	36645155	36645462	308
36,640,000	36,640,500	yes	83	500	500	91.10%	17	+	36645707	36663162	17456
		yes	83	219	500	96.30%	17	+	35221018	35221231	214
		many inter/intra									
36,640,500	36,641,000	yes	1	493	500	91.10%	17	+	36663163	36663670	508
		many inter/intra									
36,641,000	36,641,500	yes	21	451	500	91.70%	17	+	36658330	36658758	429
36,641,500	36,642,000	yes	1	500	500	92.20%	17	+	36647062	36659228	12167
		yes	1	500	500	91.10%	17	+	36658732	36664882	6151
36,642,000	36,642,500	yes	6	500	500	95.60%	17	+	36636157	36685695	49539
		yes	1	500	500	94.60%	17	+	36664883	36665383	501
		yes	6	500	500	95.90%	17	+	36659234	36675390	16157
		yes	6	500	500	94.90%	17	+	36674879	36681323	6445
		yes	1	500	500	94.20%	17	+	36647559	36648298	740
		yes	195	500	500	93.70%	17	+	36599576	36599899	324
		yes	179	500	500	90.50%	17	+	36622383	36648034	25652
		yes	291	500	500	93.10%	17	+	36600002	36659718	59717
		yes	260	498	500	92.90%	17	-	36444382	36587957	143576
		yes	260	498	500	91.10%	17	-	36436716	36533915	97200
		yes	260	498	500	94.00%	17	-	36451036	36559560	108525
		yes	301	500	500	93.20%	17	+	36600003	36622712	22710
		yes	300	414	500	90.70%	17	+	36665312	36685783	20472
36,642,500	36,643,000	yes	1	492	500	95.00%	17	+	36647936	36648541	606
		yes	1	492	500	94.80%	17	+	36636554	36637189	636
		yes	1	500	500	92.20%	17	+	36659719	36660203	485
		yes	1	500	500	92.00%	17	+	36622801	36665912	43112
		yes	1	480	500	91.80%	17	+	36599900	36681838	81939
		yes	1	483	500	90.80%	17	+	36681210	36686230	5021
		yes	18	242	500	93.00%	17	-	36527734	36587780	60047
		yes	18	243	500	90.60%	17	-	36507562	36511940	4379
		yes	133	256	500	90.20%	17	-	36559139	36577916	18778
36,643,000	36,643,500	yes	1	500	500	94.60%	17	+	36660204	36666411	6208
		yes	1	500	500	90.40%	17	+	36648550	36649048	499
		yes	1	500	500	90.40%	17	+	36637198	36637696	499
		yes	242	354	500	92.10%	17	+	36666154	36666266	113
36,643,500	36,644,000	yes	130	500	500	92.40%	17	+	36649190	36669011	19822
36,644,000	36,644,500	yes	32	495	500	92.70%	17	+	36661240	36661703	464
		yes	87	500	500	94.40%	17	+	36638294	36656178	17885
		yes	247	495	500	93.20%	17	+	36638454	36638702	249
		yes	309	495	500	96.30%	18	-	28462616	28462803	188
		yes	87	183	500	91.40%	17	+	36669097	36669197	101
36,644,500	36,645,000	yes	1	500	500	98.60%	17	+	36656179	36656683	505
		yes	1	311	500	90.70%	18	-	28462302	28462610	309
36,645,000	36,645,500	yes	1	500	500	95.20%	17	+	36656684	36657183	500
		yes	1	500	500	90.80%	17	+	36662217	36662707	491

		yes	1	500	500	91.60%	17	+	36639516	36640016	501
36,645,500	36,646,000	yes	1	500	500	95.10%	17	+	36657184	36657661	478
		yes	107	349	500	95.20%	16	-	9227181	9227633	453
		yes	116	268	500	95.10%	13	+	58134829	58135036	208
		many inter/intra									
36,646,000	36,646,500	yes	1	500	500	97.80%	17	+	36657662	36658161	500
		yes	1	483	500	91.40%	17	+	36663026	36663512	487
36,646,500	36,647,000	yes	1	500	500	98.80%	17	+	36658162	36658663	502
		yes	1	500	500	91.20%	17	+	36663530	36664033	504
		yes	24	500	500	90.40%	17	+	36640875	36641348	474
36,647,000	36,647,500	yes	1	500	500	94.40%	17	+	36658664	36659169	506
		yes	51	492	500	91.90%	17	+	36641488	36641932	445
		yes	51	500	500	92.70%	17	+	36664372	36664823	452
36,647,500	36,648,000	yes	1	500	500	96.60%	17	+	36659170	36659683	514
		yes	1	500	500	94.30%	17	+	36641941	36685645	43705
		yes	7	500	500	94.50%	17	+	36664830	36681273	16444
		yes	1	500	500	94.00%	17	+	36636093	36665333	29241
		yes	1	500	500	92.10%	17	+	36674815	36675340	526
		yes	148	500	500	94.70%	17	+	36642096	36642449	354
		yes	181	500	500	96.30%	17	+	36636283	36636617	335
		yes	245	500	500	95.20%	17	+	36599576	36599849	274
		yes	334	500	500	90.80%	17	+	36515349	36600194	84846
		yes	351	463	500	96.40%	17	+	36600003	36675624	75622
		yes	367	486	500	91.80%	17	+	36600046	36659903	59858
		yes	352	471	500	95.70%	17	+	36648052	36648306	255
		yes	367	479	500	95.60%	17	-	36587557	36587855	299
		yes	352	476	500	91.70%	17	-	36549766	36550061	296
36,648,000	36,648,500	yes	1	500	500	99.20%	17	+	36636633	36637147	515
		yes	1	500	500	96.60%	17	+	36599865	36660153	60289
		yes	1	500	500	94.60%	17	+	36659684	36665863	6180
		yes	1	500	500	92.80%	17	+	36675356	36681808	6453
		yes	10	500	500	93.70%	17	+	36600381	36676137	75757
		yes	1	500	500	91.70%	17	+	36685661	36686198	538
		yes	1	307	500	94.10%	17	+	36681289	36685616	4328
		yes	10	307	500	90.80%	17	+	36622540	36636675	14136
		yes	18	310	500	94.50%	17	-	36507305	36533833	26529
		yes	53	293	500	91.50%	17	-	36507562	36511816	4255
		yes	2	306	500	92.70%	17	+	36642379	36675325	32947
		yes	18	250	500	91.20%	17	-	36436691	36444535	7845
		yes	18	309	500	95.20%	17	-	36436677	36451111	14435
36,648,500	36,649,000	yes	1	500	500	100.00%	17	+	36637148	36637647	500
		yes	1	500	500	91.00%	17	+	36642950	36643450	501
36,649,000	36,649,500	yes	1	500	500	91.10%	17	+	36637648	36661148	23501
		yes	143	495	500	90.40%	17	+	36637789	36638142	354
36,649,500	36,650,000	many inter/intra									
36,650,000	36,650,500	many inter/intra									
36,650,500	36,651,000	many inter/intra									
36,651,000	36,651,500	many inter/intra									
36,651,500	36,652,000	many inter/intra									
36,652,000	36,652,500	many inter/intra									
36,652,500	36,653,000	many inter/intra									
36,653,000	36,653,500	many inter/intra									
36,653,500	36,654,000	many inter/intra									
36,654,000	36,654,500	many inter/intra									
36,654,500	36,655,000	many inter/intra									
36,655,000	36,655,500	many inter/intra									
36,655,500	36,656,000	many inter/intra									
36,656,000	36,656,500	yes	1	500	500	99.40%	17	+	36644320	36644820	501
		yes	1	497	500	93.20%	17	+	36661529	36662026	498
		many inter/intra									
36,656,500	36,657,000	yes	1	500	500	95.80%	17	+	36644821	36662532	17712
		yes	30	500	500	93.60%	17	+	36639054	36645315	6262
		yes	253	500	500	94.80%	17	+	36639584	36639832	249
36,657,000	36,657,500	yes	1	482	500	95.10%	17	+	36645316	36645855	540
		many inter/intra									
36,657,500	36,658,000	yes	1	500	500	98.00%	17	+	36645838	36646337	500
		yes	1	500	500	91.00%	17	+	36640201	36663365	23165
36,658,000	36,658,500	yes	1	500	500	98.60%	17	+	36646338	36646837	500
		yes	11	500	500	90.30%	17	+	36663376	36663868	493
36,658,500	36,659,000	yes	5	500	500	93.10%	17	+	36646841	36647331	491
		yes	1	496	500	91.10%	17	+	36641185	36664651	23467
		yes	1	498	500	90.00%	17	+	36663869	36684962	21094
		yes	202	497	500	92.50%	17	+	36641392	36680522	39131
36,659,000	36,659,500	yes	1	500	500	96.80%	17	+	36647221	36647830	610
		yes	1	500	500	94.30%	17	+	36641771	36681089	39319

		yes	1	500	500	94.20%	17	+	36664545	36665164	620
		yes	7	500	500	90.10%	17	+	36674650	36685476	10827
		yes	81	500	500	92.80%	17	+	36636005	36642280	6276
		yes	351	500	500	96.00%	17	+	36636284	36636433	150
		yes	376	500	500	94.40%	17	+	36675032	36675156	125
36,659,500	36,660,000	yes	1	500	500	97.60%	17	+	36636434	36648345	11912
		yes	1	500	500	97.00%	17	+	36599666	36636993	37328
		yes	1	500	500	96.40%	17	+	36642281	36665709	23429
		yes	1	500	500	95.20%	17	+	36675157	36681649	6493
		yes	12	500	500	95.40%	17	+	36622496	36642795	20300
		yes	1	467	500	95.30%	17	+	36665165	36685988	20824
		yes	1	461	500	92.50%	17	+	36681090	36681523	434
		yes	36	446	500	93.10%	17	+	36515266	36681376	166111
		yes	36	445	500	90.10%	17	-	36511662	36570322	58661
		yes	36	395	500	92.10%	17	-	36436436	36559293	122858
		yes	36	411	500	93.80%	17	-	36444503	36577761	133259
		yes	44	464	500	90.10%	17	+	36515259	36637045	121787
		yes	21	252	500	94.00%	17	+	36599977	36675366	75390
		yes	1	267	500	94.40%	17	+	36647831	36690463	42633
		yes	36	449	500	92.10%	17	-	36550053	36587855	37803
		yes	21	227	500	92.00%	17	-	36436676	36550193	113518
		yes	19	253	500	94.20%	17	+	36599828	36636557	36730
		yes	21	227	500	92.20%	17	-	36436472	36550061	113590
		yes	21	148	500	95.20%	17	-	36444520	36507778	63259
		yes	150	268	500	93.20%	17	-	36559232	36577566	18335
36,660,000	36,660,500	yes	7	500	500	93.90%	17	+	36642802	36643295	494
		yes	2	500	500	91.60%	17	+	36648347	36648845	499
		yes	2	500	500	91.60%	17	+	36636995	36637493	499
		yes	1	500	500	92.20%	17	+	36665710	36682145	16436
		yes	93	427	500	92.90%	17	+	36676076	36682071	5996
36,660,500	36,661,000	yes	1	500	500	93.20%	17	+	36643296	36649350	6055
36,661,000	36,661,500	nothing									
36,661,500	36,662,000	yes	1	497	500	92.00%	17	+	36655971	36656465	495
		yes	1	497	500	92.00%	17	+	36644291	36644786	496
		yes	1	499	500	90.00%	17	+	36638499	36638994	496
		many inter/intra									
36,662,000	36,662,500	yes	1	500	500	92.90%	17	+	36656470	36656966	497
		yes	1	500	500	90.80%	17	+	36644791	36645282	492
		yes	1	499	500	90.10%	17	+	36638996	36639798	803
36,662,500	36,663,000	many inter/intra									
36,663,000	36,663,500	yes	1	499	500	90.40%	17	+	36657636	36658131	496
		yes	1	499	500	90.40%	17	+	36645974	36646469	496
		yes	170	414	500	91.60%	1	-	108657572	108657832	261
36,663,500	36,664,000	yes	55	500	500	90.10%	17	+	36640876	36684586	43711
36,664,000	36,664,500	many inter/intra									
36,664,500	36,665,000	yes	1	500	500	93.00%	17	+	36658846	36685311	26466
		yes	1	500	500	92.90%	17	+	36647176	36680924	33749
		yes	2	500	500	91.70%	17	+	36641617	36642115	499
		yes	20	500	500	91.00%	17	+	36635785	36636268	484
36,665,000	36,665,500	yes	1	500	500	97.00%	17	+	36636269	36636783	515
		yes	1	500	500	96.80%	17	+	36642116	36675506	33391
		yes	1	499	500	95.60%	17	+	36647666	36685810	38145
		yes	10	499	500	94.90%	17	+	36680934	36681438	505
		yes	1	500	500	94.10%	17	+	36659336	36659804	469
		yes	79	500	500	95.50%	17	+	36599576	36642585	43010
		yes	63	500	500	90.30%	17	+	36622383	36648135	25753
		yes	10	441	500	93.40%	17	+	36675000	36690484	15485
		yes	139	436	500	90.80%	17	-	36549817	36570502	20686
		yes	198	436	500	91.60%	17	-	36511722	36577854	66133
		yes	186	423	500	94.50%	17	+	36599977	36648015	48039
		yes	186	385	500	90.30%	17	+	36515371	36648299	132929
		yes	202	451	500	93.00%	17	-	36549910	36570306	20397
		yes	186	412	500	91.70%	17	+	36600061	36648296	48236
		yes	258	410	500	93.90%	17	-	36444369	36511940	67572
		yes	186	382	500	94.20%	17	+	36648052	36659881	11830
36,665,500	36,666,000	yes	1	475	500	93.30%	17	+	36636784	36676247	39464
		yes	1	459	500	94.40%	17	+	36648136	36660249	12114
		yes	1	460	500	92.20%	17	+	36642586	36681903	39318
		yes	1	456	500	92.80%	17	+	36659805	36686289	26485
		yes	1	264	500	91.60%	17	+	36681440	36681710	271
		yes	1	177	500	93.30%	17	+	36685812	36685988	177
		yes	1	171	500	92.40%	17	+	36675507	36685703	10197
		yes	53	174	500	96.00%	17	-	36507305	36570351	63047
		yes	53	161	500	93.60%	17	-	36507528	36527648	20121
		yes	61	170	500	91.00%	17	+	36636565	36675325	38761

		yes	49	156	500	92.20%	17	-	36559228	36559509	282
		yes	53	173	500	93.60%	17	-	36436707	36507447	70741
		yes	66	170	500	93.20%	17	-	36511632	36587856	76225
36,666,000	36,666,500	nothing									
36,666,500	36,667,000	many inter/intra									
36,667,000	36,667,500	many inter/intra									
36,667,500	36,668,000	many inter/intra									
36,668,000	36,668,500	nothing									
36,668,500	36,669,000	many inter/intra									
36,669,000	36,669,500	nothing									
36,669,500	36,670,000	many inter/intra									
36,670,000	36,670,500	many inter/intra									
36,670,500	36,671,000	many inter/intra									
36,671,000	36,671,500	many inter/intra									
36,671,500	36,672,000	many inter/intra									
36,672,000	36,672,500	many inter/intra									
36,672,500	36,673,000	nothing									
36,673,000	36,673,500	many inter/intra									
36,673,500	36,674,000	nothing									
36,674,000	36,674,500	nothing									
36,674,500	36,675,000	yes	61	313	500	95.10%	6	+	170184707	170185274	568
36,675,000	36,675,500	yes	315	467	500	92.90%	17	+	36636092	36636244	153
36,675,500	36,676,000	yes	1	500	500	95.40%	17	+	36636278	36636776	499
		yes	1	500	500	95.40%	17	+	36642125	36665492	23368
		yes	1	494	500	93.60%	17	+	36680934	36681426	493
		yes	1	494	500	93.30%	17	+	36685321	36685798	478
		yes	1	500	500	90.80%	17	+	36647675	36648128	454
		yes	72	500	500	94.60%	17	+	36599577	36642578	43002
		yes	55	500	500	92.50%	17	+	36622383	36659797	37415
		yes	1	448	500	90.30%	17	+	36665009	36690484	25476
		yes	179	474	500	91.10%	17	+	36599831	36659864	60034
		yes	190	444	500	92.70%	17	+	36515263	36659945	144683
		yes	190	443	500	93.20%	17	-	36507534	36559461	51928
		yes	179	399	500	93.80%	17	+	36599978	36659888	59911
		yes	264	469	500	90.50%	17	+	36482025	36600292	118268
		yes	179	399	500	91.90%	17	-	36444372	36533587	89216
		yes	190	441	500	91.60%	17	-	36577444	36587858	10415
		yes	179	443	500	92.00%	17	-	36533764	36550192	16429
		yes	265	440	500	95.10%	17	+	36515446	36648098	132653
		yes	190	386	500	94.40%	17	-	36559151	36570385	11235
		yes	284	445	500	95.10%	17	-	36587579	36594283	6705
		yes	336	458	500	93.00%	17	-	36570261	36577762	7502
		yes	190	300	500	94.70%	17	-	36533523	36559281	25759
		yes	194	300	500	92.60%	17	-	36570077	36570306	230
36,676,000	36,676,500	many inter/intra									
36,676,500	36,677,000	nothing									
36,677,000	36,677,500	yes	397	500	500	92.90%	17	+	36638421	36638524	104
36,677,500	36,678,000	many inter/intra									
36,678,000	36,678,500	nothing									
36,678,500	36,679,000	many inter/intra									
36,679,000	36,679,500	nothing									
36,679,500	36,680,000	nothing									
36,680,000	36,680,500	yes	1	500	500	92.10%	17	+	36684432	36684938	507
		yes	1	359	500	92.10%	17	+	36658466	36674470	16005
		yes	1	478	500	91.00%	17	+	36646804	36647283	480
36,680,500	36,681,000	nothing									
36,681,000	36,681,500	yes	1	500	500	97.40%	17	+	36659410	36685871	26462
		yes	1	499	500	95.20%	17	+	36665075	36675565	10491
		yes	1	500	500	95.00%	17	+	36636344	36636843	500
		yes	4	500	500	95.80%	17	+	36599576	36648195	48620
		yes	1	499	500	93.80%	17	+	36647741	36665558	17818
		yes	1	500	500	93.20%	17	+	36642191	36659864	17674
		yes	45	413	500	95.50%	17	+	36622439	36659882	37444
		yes	126	412	500	92.80%	17	+	36515266	36648296	133031
		yes	64	417	500	90.30%	17	-	36436681	36570502	133822
		yes	1	414	500	92.80%	17	+	36685387	36690559	5173
		yes	126	407	500	91.50%	17	-	36533643	36577671	44029
		yes	126	413	500	90.50%	17	-	36559118	36587870	28753
		yes	64	417	500	91.80%	17	-	36444485	36533933	89449
		yes	195	485	500	92.50%	17	+	36481994	36622678	140685
		yes	127	414	500	92.50%	17	-	36549959	36570306	20348
		yes	126	414	500	91.60%	17	-	36570115	36577761	7647
		yes	126	332	500	92.80%	17	-	36436676	36559293	122618
		yes	126	376	500	91.50%	17	-	36511662	36570382	58721
		yes	134	406	500	93.00%	17	+	36636885	36659944	23060

		yes	111	252	500	93.60%	17	-	36436744	36550193	113450
		yes	126	318	500	94.50%	17	-	36511642	36533813	22172
		yes	212	412	500	93.80%	17	+	36515550	36600146	84597
		yes	134	238	500	94.60%	17	-	36444520	36550185	105666
36,681,500	36,682,000	yes	1	494	500	92.70%	17	+	36685872	36686383	512
		yes	1	498	500	90.60%	17	+	36665560	36676327	10768
		yes	1	403	500	91.00%	17	+	36648091	36660249	12159
36,682,000	36,682,500	yes	1	500	500	91.80%	17	+	36686393	36686902	510
36,682,500	36,683,000	yes	1	499	500	93.70%	17	+	36686903	36687403	501
		yes	83	363	500	91.00%	17	+	36677606	36677916	311
36,683,000	36,683,500	yes	1	500	500	91.70%	17	+	36687405	36687910	506
36,683,500	36,684,000	yes	287	488	500	90.00%	11	+	45524584	45524821	238
36,684,000	36,684,500	nothing									
36,684,500	36,685,000	yes	1	500	500	90.00%	17	+	36680066	36680560	495
36,685,000	36,685,500	yes	1	500	500	90.10%	17	+	36641804	36642303	500
		yes	339	500	500	92.60%	17	+	36675018	36675179	162
		yes	387	500	500	90.70%	17	+	36599573	36599688	116
36,685,500	36,686,000	yes	1	486	500	97.00%	17	+	36681113	36681613	501
		yes	1	494	500	96.20%	17	+	36599689	36648318	48630
		yes	1	494	500	95.80%	17	+	36636457	36636966	510
		yes	1	489	500	94.90%	17	+	36665188	36665676	489
		yes	1	489	500	94.70%	17	+	36642304	36659966	17663
		yes	1	472	500	92.60%	17	+	36675180	36675624	445
		yes	1	489	500	92.20%	17	+	36515374	36642762	127389
		yes	1	396	500	96.90%	17	+	36647854	36648306	453
		yes	10	475	500	90.20%	17	+	36515263	36623011	107749
		yes	1	245	500	98.30%	17	+	36659523	36659767	245
		yes	10	477	500	92.40%	17	-	36533690	36577854	44165
		yes	1	428	500	90.70%	17	+	36622508	36648044	25537
		yes	10	426	500	91.10%	17	-	36436829	36587858	151030
		yes	12	426	500	91.50%	17	-	36444495	36577762	133268
		yes	1	284	500	90.00%	17	+	36599980	36659716	59737
		yes	95	467	500	92.50%	17	-	36577637	36594448	16812
		yes	1	282	500	91.40%	17	-	36436673	36507775	71103
		yes	1	222	500	92.80%	17	-	36451023	36550058	99036
		yes	1	387	500	90.40%	17	-	36444477	36511783	67307
		yes	88	426	500	91.00%	17	+	36515419	36600195	84777
		yes	10	112	500	94.70%	17	+	36665326	36665662	337
		yes	230	477	500	92.00%	11	-	1599320	1599769	450
36,686,000	36,686,500	yes	16	500	500	90.30%	17	+	36681643	36682106	464
		yes	179	494	500	90.50%	17	+	36637127	36660454	23328
		yes	179	454	500	92.40%	17	+	36648479	36648761	283
36,686,500	36,687,000	yes	1	500	500	91.40%	17	+	36682107	36682596	490
36,687,000	36,687,500	yes	1	500	500	91.20%	17	+	36682603	36683094	492
36,687,500	36,688,000	nothing									
36,688,000	36,688,500	yes	1	266	500	92.10%	17	+	36683589	36688731	5143
36,688,500	36,689,000	many inter/intra									
36,689,000	36,689,500	many inter/intra									
36,689,500	36,690,000	many inter/intra									
36,690,000	36,690,500	nothing									
36,690,500	36,691,000	nothing									
36,691,000	36,691,500	many inter/intra									
36,691,500	36,692,000	nothing									
36,692,000	36,692,500	nothing									
36,692,500	36,693,000	nothing									
36,693,000	36,693,500	nothing									
36,693,500	36,694,000	nothing									
36,694,000	36,694,500	nothing									
36,694,500	36,695,000	nothing									
36,695,000	36,695,500	nothing									
36,695,500	36,696,000	nothing									
36,696,000	36,696,500	many inter/intra									
36,696,500	36,697,000	many inter/intra									
36,697,000	36,697,500	many inter/intra									
36,697,500	36,698,000	many inter/intra									
36,698,000	36,698,500	many inter/intra									
36,698,500	36,699,000	many inter/intra									
36,699,000	36,699,500	nothing									
36,699,500	36,700,000	many inter/intra									
36,700,000	36,700,500	many inter/intra									
36,700,500	36,701,000	nothing									
36,701,000	36,701,500	nothing									
36,701,500	36,702,000	yes	225	500	500	97.7	1	-	228786243	228786611	369
36,702,000	36,702,500	many inter/intra									
36,702,500	36,703,000	many inter/intra									
36,703,000	36,703,500	many inter/intra									

36,703,500	36,704,000	yes	1	172	500	95.20%	12	-	110626136	110626361	226
36,704,000	36,704,500	nothing									
36,704,500	36,705,000	many inter/intra									
36,705,000	36,705,500	many inter/intra									
36,705,500	36,706,000	many inter/intra									
36,706,000	36,706,500	many inter/intra									
36,706,500	36,707,000	nothing									
36,707,000	36,707,500	nothing									
36,707,500	36,708,000	nothing									
36,708,000	36,708,500	many inter/intra									
36,708,500	36,709,000	many inter/intra									
36,709,000	36,709,500	nothing									
36,709,500	36,710,000	nothing									
36,710,000	36,710,500	many inter/intra									
36,710,500	36,711,000	many inter/intra									
36,711,000	36,711,500	nothing									
36,711,500	36,712,000	nothing									
36,712,000	36,712,500	nothing									
36,712,500	36,713,000	nothing									
36,713,000	36,713,500	nothing									
36,713,500	36,714,000	nothing									
36,714,000	36,714,500	yes	265	438	500	95.8	15	-	94768297	94768563	267
36,714,500	36,715,000	nothing									
36,715,000	36,715,500	many inter/intra									
36,715,500	36,716,000	many inter/intra									
36,716,000	36,716,500	nothing									
36,716,500	36,717,000	nothing									
36,717,000	36,717,500	nothing									
36,717,500	36,718,000	nothing									
36,718,000	36,718,500	nothing									
36,718,500	36,719,000	nothing									
36,719,000	36,719,500	nothing									
36,719,500	36,720,000	many inter/intra									
36,720,000	36,720,500	many inter/intra									
36,720,500	36,721,000	nothing									
36,721,000	36,721,500	many inter/intra									
36,721,500	36,722,000	nothing									
36,722,000	36,722,500	nothing									
36,722,500	36,723,000	nothing									
36,723,000	36,723,500	many inter/intra									
36,723,500	36,724,000	nothing									
36,724,000	36,724,500	nothing									
36,724,500	36,725,000	many inter/intra									
36,725,000	36,725,500	nothing									
36,725,500	36,726,000	yes	239	366	500	97	10	-	4939868	4940161	294
36,726,000	36,726,500	nothing									
36,726,500	36,727,000	many inter/intra									
36,727,000	36,727,500	nothing									
36,727,500	36,728,000	nothing									
36,728,000	36,728,500	nothing									
36,728,500	36,729,000	nothing									
36,729,000	36,729,500	nothing									
36,729,500	36,730,000	many inter/intra									
36,730,000	36,730,500	many inter/intra									
36,730,500	36,731,000	many inter/intra									
36,731,000	36,731,500	many inter/intra									
36,731,500	36,732,000	nothing									
36,732,000	36,732,500	many inter/intra									
36,732,500	36,733,000	nothing									
36,733,000	36,733,500	nothing									
36,733,500	36,734,000	nothing									
36,734,000	36,734,500	nothing									
36,734,500	36,735,000	nothing									
36,735,000	36,735,500	nothing									
36,735,500	36,736,000	many inter									
36,736,000	36,736,500	nothing									
36,736,500	36,737,000	many inter									
36,737,000	36,737,500	many inter									
36,737,500	36,738,000	many inter									
36,738,000	36,738,500	yes	1	500	500	90.1	17	+	22781421	22781955	535
36,738,500	36,739,000	many inter/intra	1	498	500	91.3	17	+	22781956	22782533	578
36,739,000	36,739,500	many inter/intra									
36,739,500	36,740,000	many inter/intra									
36,740,000	36,740,500	many inter/intra									
36,740,500	36,741,000	many inter/intra									
36,741,000	36,741,500	nothing									
36,741,500	36,742,000	nothing									

36,742,000	36,742,500	many inter/intra									
36,742,500	36,743,000	nothing									
36,743,000	36,743,500	nothing									
36,743,500	36,744,000	yes	143	500	500	90.1	17	-	33356798	33357176	379
		yes	143	500	500	90.1	17	+	33553413	33553791	379
36,744,000	36,744,500	many inter/intra									
36,744,500	36,745,000	nothing									
36,745,000	36,745,500	nothing									
36,745,500	36,746,000	many inter/intra									
36,746,000	36,746,500	nothing									
36,746,500	36,747,000	many inter/intra									
36,747,000	36,747,500	nothing									
36,747,500	36,748,000	yes	87	220	500	91.3	17	-	36747586	36747719	134
36,748,000	36,748,500	yes	361	500	500	90.8	17	+	36747665	36747805	141
		yes	1	103	500	97.1	X	+	35098501	35098603	103
		yes	166	315	500	97.3	16	+	79282456	79282955	500
36,748,500	36,749,000	nothing									
36,749,000	36,749,500	nothing									
36,749,500	36,750,000	nothing									

Table S2. LD Map Boundaries

Population	# of SNPs ($r^2 \geq 0.9$)	Range of SNPs ($r^2 \geq 0.9$)	Haploblock Length (kb)
**YRI**	17	36,653,092-36,718,262	65.17
**CEU**	27	36,647,015-36,761,496	87.48
**CHB+JPT**	31	36,548,130-36,784,735	236.61

Population	# of SNPs ($r^2 \geq 0.8$)	Range of SNPs ($r^2 \geq 0.8$)	Haploblock Length (kb)
**YRI**	20	36,567,324-36,718,262	150.94
**CEU**	33	36,606,030-36,784,735	178.71
**CHB+JPT**	40	36,548,130-36,784,735	236.61

Table S3. Cancer Gene amplification and complex genomic regions

Gene/Region	Region Coordinates	Cytoband	Cancer	Gaps Coordinates	SD Coordinates
ERBB2	35109780-35138441	17q12-	Breast		36400000-36700000
		17q21.2			
CCND1	69165054-69178423	11q13	Breast	68846378-68848982	67188422-67546827
				69437112-69454899	70914582-71302090
1q21	142400001-148000000	1q21	HCC	142386049-142436048	142400001-148114553
			Breast	142562526-142612525	
			Bladder	142807141-142857140	
			Nasopharynk	142935839-142985838	
			Esophagus	143113102-143163101	
				143333771-143383770	
				143422082-143472081	
				143472082-143522081	
				144544476-144594475	
				144876008-144926007	
				146492663-146542662	
				146727983-146777982	
				146950772-147000771	
				147221085-147271084	
				147726270-147776269	
MYCL1	40137873-40140274	1p34	Lung		39728394-40134820
			Medulloblastoma		
MDM4	202760991-202794811	1q32.1	Retinoblastoma	204189331-204239330	204239332-204356320
				204498845-204548844	204548846-204649960
MycN	15999497-16004580	2p24	Neuroblastoma	16171404-16221403	

HCC - Hepatocellular Carcinoma

SD - Segmental Duplication

Table S4. HapMap Sample ID List

ID	Population	Family	Relationship
NA18517	YRI		
NA18507	YRI		
NA18956	JPT		
NA19240	YRI		
NA18555	CHB		
NA12878	CEU		
NA19129	YRI		
NA12156	CEU		
NA12248	CEU	1	Father
NA12249	CEU		Mother
NA10835	CEU		Child
NA07022	CEU	2	Father
NA07056	CEU		Mother
NA07019	CEU		Child
NA12005	CEU	3	Father
NA12006	CEU		Mother
NA10839	CEU		Child
NA12144	CEU	4	Father
NA12145	CEU		Mother
NA10846	CEU		Child
NA07034	CEU	5	Father
NA07055	CEU		Mother
NA07048	CEU		Child
NA07357	CEU	6	Father
NA07345	CEU		Mother
NA07348	CEU		Child
NA12043	CEU	7	Father
NA12044	CEU		Mother
NA10857	CEU		Child
NA11881	CEU	8	Father
NA11882	CEU		Mother
NA10859	CEU		Child
NA11829	CEU	9	Father
NA11830	CEU		Mother
NA10856	CEU		Child
NA11994	CEU	10	Father
NA11995	CEU		Mother
NA10861	CEU		Child

Table S5. PCR and qPCR Primer List

Primer	Forward/Reverse	Sequence 5' to 3'	Experiment	Figure
ERBB2F-35125941	Forward	GAACTCTTCCTCTCCCTACATCGG	qPCR	3
ERBB2R-35126547	Reverse	CCCTTTTCTGCCACTCTGTTGC	qPCR	3
1F-35689009	Forward	TGCTCCAGGATAACAAAGAAGGG	qPCR	3
1R-35689139	Reverse	AGGAAGAGTCTGCTCAGTCAGGTG	qPCR	3
2F-35700826	Forward	TGTGTTTCAGGCGATGACAACC	qPCR	3
2R-35700930	Reverse	AAGTGTATGTGAGTGAGGGGGACC	qPCR	3
3F-35991666	Forward	CTCTGGACTTGTCATTTAGTGGGC	qPCR	3
3R-35991796	Reverse	GTGGAGGCTCATTTTCGTTGG	qPCR	3
4F-36322319	Forward	GAATGCCCTTCTTTGGTCTGC	qPCR	3
4R-36322516	Reverse	GCCTCACACCCTTCTCAAGTATCC	qPCR	3
5F-36376257	Forward	AGATTCCAGTTCAGCATTCTCTCG	qPCR	3
5R-36376456	Reverse	AACCCACTCCTCGCTTTTGTCG	qPCR	3
6F-36544207	Forward	CGCTGTTTGGGCAGATTGTTC	qPCR	3
6R-36544337	Reverse	GGCAGACTAAGGTTTTCTTGTCTCC	qPCR	3
7F-36695145	Forward	ACACCACATACTTCTTCCTTCCTCC	qPCR	3
7R-36695329	Reverse	GGAAACACAGGCTGGTTTTTCTG	qPCR	3
Control F	Forward	TGGGCTCAAGTTCTTTGGGGTC	qPCR	3
Control R	Reverse	CGCTGTGGCTGATGTGTAGTAGAG	qPCR	3
17-36675156	Forward	ATGACCCACTGTTGCTCCCC	PCR	6
17-36681634	Reverse	CAGAAGGTTGCTGTGTGAGGATG	PCR	6
17-36681089	Forward	ATGACCCACTGCTGTTCCCC	PCR	6
